# GM-CSF^+^ Th: a central player in autoimmunity

**DOI:** 10.7150/thno.121766

**Published:** 2025-09-12

**Authors:** Sha-Sha Fan, Xuan Xu, Yu-Bin Luo, Xiang-Yu Meng, Yi Liu

**Affiliations:** 1 Department of Rheumatology and Immunology, Laboratory of Rheumatology and Immunology, West China Hospital, Sichuan University, Chengdu 610041, China.; 2 Hubei Provincial Key Laboratory of Occurence and Intervention of Rheumatic Diseases, Hubei Minzu University, Enshi 445000, China.; 3 Institute of Immunology and Inflammation, Frontiers Science Center for Disease-Related Molecular Network, West China Hospital, Chengdu 610041, China.; 4 Health Science Center, Hubei Minzu University, Enshi 445000, China.; 5 School of Life Sciences, Anhui Medical University, Hefei 230032, China.; 6 West China Lecheng Hospital, Sichuan University, Boao 571435, Hainan, China.

**Keywords:** GM-CSF^+^ Th cells, autoimmune diseases, GM-CSF, differentiation regulation, pathogenic mechanisms, clinical applications

## Abstract

Autoimmune diseases are driven by a breach of self-tolerance, leading to chronic inflammation and organ damage. While the Th1 and Th17 paradigms have long dominated our understanding of T-cell-mediated pathology, a distinct subset of CD4^+^ T helper cells defined by the production of granulocyte-macrophage colony-stimulating factor (GM-CSF) has emerged as a central and non-redundant pathogenic driver. These GM-CSF-producing Th cells orchestrate autoimmune pathology across a spectrum of diseases, including multiple sclerosis, rheumatoid arthritis, and type 1 diabetes, among others. Mechanistically, they act as critical amplifiers of inflammation by activating and recruiting myeloid cells, which in turn mediate tissue destruction. Furthermore, they establish vicious feedback loops, enhancing their own differentiation and promoting the function of other effector T cells. Despite their established importance, significant heterogeneity and plasticity exist in their phenotype and regulatory networks, with context-dependent variations across different diseases and species. This review synthesizes our current understanding of these pivotal cells, critically evaluating their classification, molecular identity, and the signaling pathways governing their differentiation. We further dissect their specific roles in major autoimmune diseases and provide a forward-looking perspective on their immense translational potential, from novel therapeutic strategies targeting the GM-CSF axis to their use as biomarkers for patient stratification and monitoring.

## 1. Introduction

Autoimmune diseases (AIDs) are chronic inflammatory conditions characterized by aberrant immune responses against self-tissues. They encompass a broad range of conditions, including multiple sclerosis (MS), rheumatoid arthritis (RA), type 1 diabetes (T1D), and inflammatory bowel disease (IBD). The pathogenesis of AIDs is driven by complex interactions between genetic susceptibility, environmental triggers, and immune dysregulation, although the precise molecular mechanisms remain incompletely understood 1. Epidemiological evidence suggests that approximately 7.6%-10.2% of the global population suffers from AIDs, which significantly impairs quality of life and imposes a growing economic burden on healthcare systems 2. The incidence of these diseases continues to rise, and the absence of effective early prevention strategies, combined with limited public awareness, further complicates disease management 3.

Within the immune regulatory network of AIDs, abnormal activation of CD4^+^ T helper (Th) cells represents a key driving factor. Naive CD4^+^ T cells differentiate into various subsets, including Th1, Th2, and Th17, as well as regulatory T cells (Tregs), under the influence of antigenic stimulation, changes in cytokine microenvironments, and transcription factor-driven regulation. These subsets mediate pro-inflammatory or anti-inflammatory responses by secreting specific cytokines (e.g., IFN-γ/TNF-α from Th1 cells, IL-17 from Th17 cells) 4, 5. Disruption of this balance contributes to persistent inflammation and immune-mediated tissue damage, which are central features of autoimmune disease pathophysiology. In particular, hyperactivation of Th1 and/or Th17 cells has been implicated as a key mechanism of disease progression in several AIDs, including MS, RA, and T1D 6, 7.

However, recent research has revealed contradictory phenomena and challenges the long established Th1/Th17 dogma. In mouse models of AIDs such as MS and T1D, among others, knockout of key factors including IFN-γ, IL-17, T-bet, Rel, or RORγt does not consistently attenuate disease progression and may even exacerbate it. These findings suggest that disease pathogenesis is not solely dependent on canonical Th1 or Th17 pathways, but rather reflects a context-dependent interplay of effector T cell subsets with compensatory mechanisms and functional plasticity 8-18. This finding suggests that the pathogenic role of the traditional Th1/Th17 axis may involve functional redundancy or microenvironment dependency, prompting researchers to reconsider the potential regulatory roles of other Th subsets in AIDs.

In recent years, the landscape of Th cell biology has been reshaped by the emergence of a distinct lineage defined by its production of GM-CSF. While GM-CSF can be secreted by various cell types, compelling evidence from multiple AIDs indicates that CD4^+^ T cells are the predominant source of this cytokine in both peripheral blood and inflamed tissues. This pathogenic Th subset was first formally identified in 2013 from *in vitr*o differentiation experiments, which revealed a population characterized by abundant GM-CSF secretion but minimal expression of canonical lineage-defining cytokines (e.g., IFN-γ, IL-17) or transcription factors (e.g., T-bet, RORγt). This as a result has initially led to its designation as a potentially independent lineage, provisionally named “ThGM” 19.

However, the identity of these cells is complicated by their profound plasticity. *In vivo*, GM-CSF is often co-expressed with other pro-inflammatory molecules like TNF-α; and under the influence of cytokines such as IL-12, these cells can be induced to acquire a Th1-like, IFN-γ-producing phenotype. This functional flexibility, combined with a lack of unique surface markers or a stable transcriptional signature, has invoked a long-standing controversy regarding their precise definition: Are they a stable, independent lineage, or a transient, highly activated state that other Th subsets can adopt? This ambiguity has resulted in a plethora of overlapping terminologies in the literature (**Table 1**), necessitating a clear and unified nomenclature. For the purpose of this review, we will use the encompassing term “GM-CSF-producing Th cells” (i.e., GM-CSF^+^ Th cells) to refer to any CD4^+^ T cell for which GM-CSF production is a key functional feature.

Regardless of their classification, their pathogenic importance is undisputed. Functional studies have revealed that GM-CSF^+^ Th cells act as powerful “amplifiers” of immune responses. They engage in autocrine feed-forward loops where GM-CSF promotes its own expression, and they participate in cross-regulatory networks that enhance the function of other effector T cell subsets 19, 20. Their indispensable role was definitively demonstrated in preclinical models of autoimmunity, such as experimental autoimmune encephalomyelitis (EAE). Genetic deletion of *Csf2* (the gene encoding GM-CSF) specifically in CD4^+^ T cells renders mice resistant to EAE, and this protection is reversed by the adoptive transfer of GM-CSF^+^ Th cells, confirming their non-redundant pathogenic function 21.

**Figure 1:**
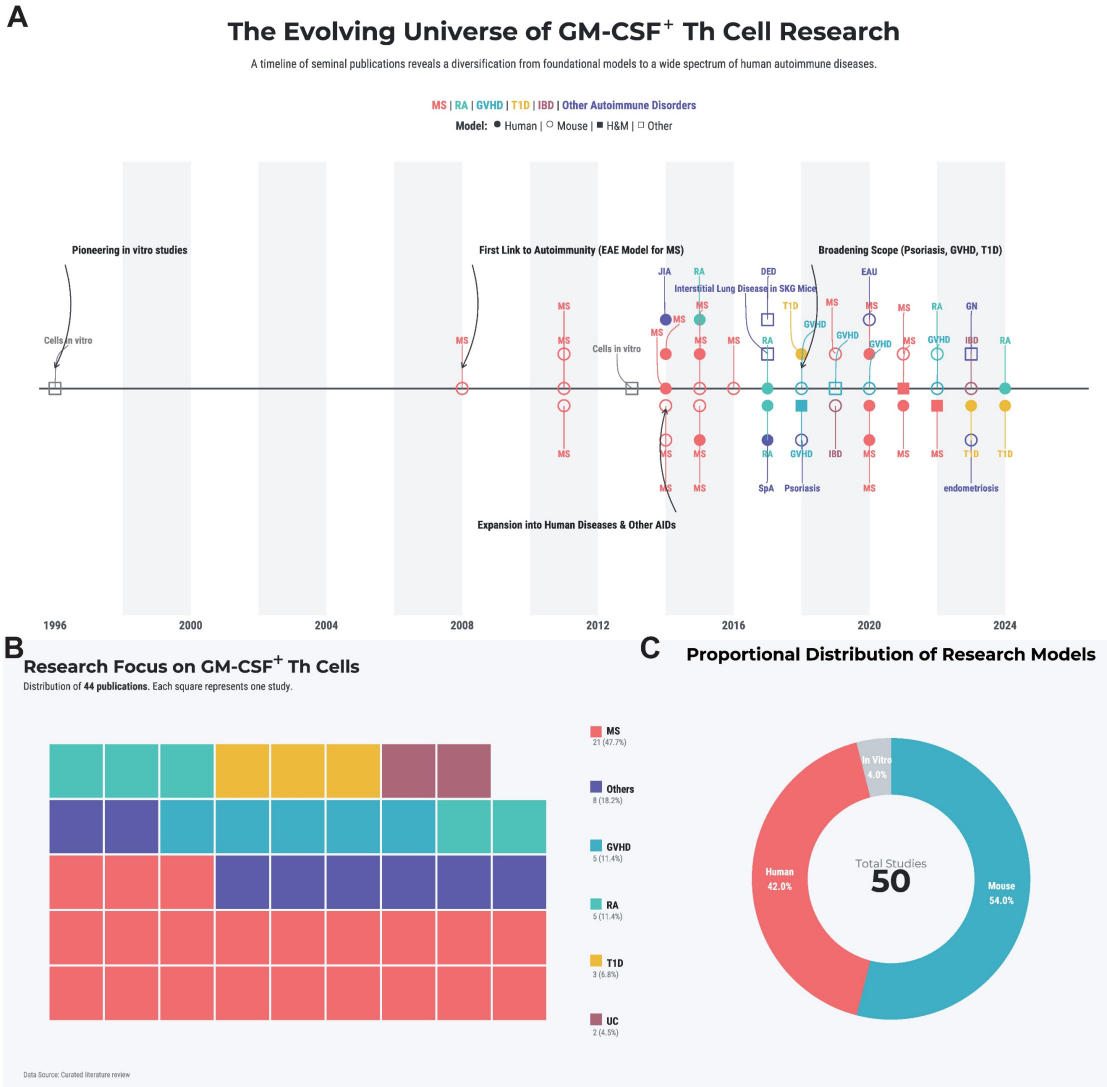
**Literature landscape of GM-CSF^+^ Th cells in autoimmunity. (A)** The historical trend of publications, with key landmark studies highlighted by black arrows. **(B)** A treemap illustrating the spectrum of autoimmune diseases studied in the literature. Each colored square denotes a single study, categorized by disease. **(C)** Distribution of study subject types. The area of each circle is proportional to the number of studies conducted on that subject type. Abbreviations: DED, dry eye disease; EAU, experimental autoimmune uveoretinitis; GN, glomerulonephritis; GVHD, graft-versus-host disease; ILD, interstitial lung disease; JIA, juvenile idiopathic arthritis; MS, mutiple sclerosis; RA, rheumatoid arthritis; SpA, spondyloarthritis; T1D, type 1 diabetes; UC, ulcerative colitis.

This review will systematically dissect the biology of these pivotal cells. We will synthesize the current understanding of their molecular identity and the complex regulatory networks governing their differentiation. Furthermore, we will analyze their specific pathogenic roles across the spectrum of major autoimmune diseases and, based on this evidence, critically evaluate emerging therapeutic strategies that target this axis. By integrating foundational research with translational prospects, we aim to provide a comprehensive theoretical framework for the future of precision intervention in autoimmunity. A landscape summary of the systematically retrieved literature is provided in **Figure 1A-C** and **Table S1**.

## 2. Molecular Characteristics and Differentiation Regulation of GM-CSF^+^ Th Cells

**Figure 2:**
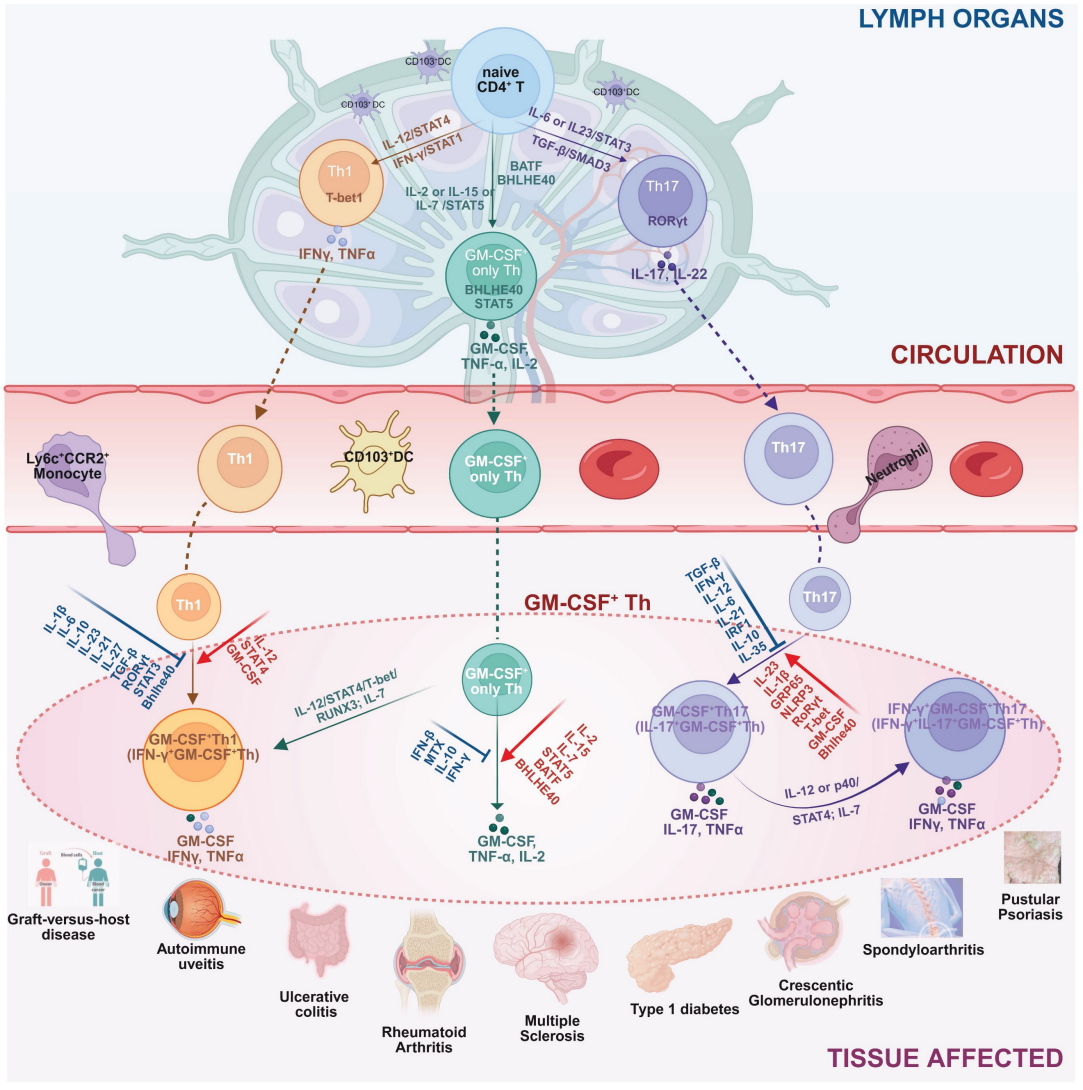
** The differentiation pathway, subtype heterogeneity, and phenotype plasticity of GM-CSF^+^ Th cells, plus a collection of related driving and suppressing factors, as well as associated diseases.** Illustrated in the diagram the generation and functional specialization of GM-CSF⁺ Th cells, beginning in the lymph organs where naive CD4⁺ T cells differentiate into Th1, Th17, or “GM-CSF only” Th lineages, driven by distinct cytokine signals (e.g., IL-12, IL-6, IL-2). After entering circulation, these cells migrate to peripheral tissues where they exhibit significant plasticity. In the inflammatory tissue microenvironment, Th1 and Th17 cells can be induced to produce GM-CSF, becoming pathogenic GM-CSF⁺ Th1 and GM-CSF⁺ Th17 cells. A further shift can occur where GM-CSF⁺ Th17 cells also gain the ability to produce IFN-γ, creating a highly inflammatory hybrid phenotype. Shown the specific cytokines and transcription factors that promote (red arrows) or suppress (blue T-bars) these states, and the resulting pathogenic cell subtypes were linked to a spectrum of human inflammatory and autoimmune diseases, including multiple sclerosis, rheumatoid arthritis, and psoriasis. Created in BioRender. Meng, X. (2025) https://BioRender.com/o6m32us.

The complex molecular aspects of GM-CSF^+^ Th cells, including differentiation pathway, subtype heterogeneity, and plasticity of GM-CSF^+^ Th cells, plus a collection of related driving and suppressing factors, as well as associated diseases, are graphically summarized in **Figure 2**.

### 2.1 Molecular diversity and disease specificity of GM-CSF^+^ Th cells

GM-CSF^+^ Th cells represent a highly heterogeneous subset of helper T cells whose molecular characteristics exhibit significant disease-specific characteristics despite sharing common features. All GM-CSF^+^ Th cell subsets can be defined by high expression of GM-CSF as their core characteristic, while frequently co-expressing TNF-α, IL-2, and IL-3. Some subsets may further co-express IFN-γ or IL-17A, indicating diverse pro-inflammatory effects when sub-grouped per common pathogenic factors 14, 22, 23. Categorizations of GM-CSF^+^ Th cells vary across studies, but based on cellular characteristics, GM-CSF^+^ Th cells primarily fall within four subtypes, namely the GM-CSF^+^ only Th cells, GM-CSF^+^IFN-γ^+^ Th cells (i.e., the Th1-like), GM-CSF^+^IL-17^+^ Th cells (i.e., the Th17-like), and GM-CSF^+^IFN-γ^+^IL-17^+^ Th cells (i.e., the triple-positive or Th1/Th17-like).

In humans, Th1-like cells constitute the dominant GM-CSF⁺ Th subset. IL-12 promotes *GM-CSF* expression in these cells, while IL-1β, IL-6, and IL-23 may exert context-dependent suppressive effects on GM-CSF secretion, potentially as part of feedback or resolution mechanisms in inflammatory environments. Previous studies have revealed that the transcription factor regulatory network involved in autoimmune diseases exhibits several conserved features, prominently including members of the STAT family (e.g., *STAT3, STAT4*, and *STAT5*), the RUNX family (e.g., *RUNX1* and *RUNX3*), and BHLHE40. Notably, BHLHE40 has been shown to directly bind to the *CSF2* promoter, thereby promoting *GM-CSF* expression, while the RUNX family plays a key role in regulating cellular plasticity, enabling immune cells to transition between functional states relevant to autoimmune pathogenesis 24-26. Notably, genome-wide chromatin accessibility analysis has revealed unique DNA accessibility regions and gene expression patterns in GM-CSF^+^ Th cells, suggesting epigenetic determinants in their differentiation 27. Additionally, DNA methylation analysis shows that hypomethylation of the GM-CSF promoter region promotes GM-CSF secretion in GM-CSF^+^ Th cells, further suggesting the critical regulatory role of epigenetic modifications in determining the GM-CSF^+^ Th phenotype 27. From a translational perspective, these findings provide a theoretical basis for developing targeted therapies through epigenetic regulation, suggesting possible effects of DNA methyltransferase inhibitors or histone deacetylase inhibitors.

Regarding disease specificity, GM-CSF^+^ Th cells may exhibit differential characteristics in various pathological backgrounds. For example, GM-CSF^+^ Th cells in MS and type 1 diabetes (T1D) often co-express both the Th1 (e.g., T-bet and CXCR3) and Th17 (e.g., RORγt and CCR6) markers, forming the so-called “Th1/Th17 hybrid subsets”, namely the triple-positive phenotype (IFN-γ^+^IL-17A^+^GM-CSF^+^) 13, 22, 28. In contrast, GM-CSF^+^ Th cells in RA basically manifest the Th1-like characteristics, co-expressing IFN-γ^+^ and TNF-α^+^, while in certain cases become negative in Th1 and Th17 markers but exclusively secretes GM-CSF and IL-21 29. Particularly, GM-CSF^+^ Th cells in IBD predominantly exhibit a single GM-CSF^+^ phenotype without expressing any markers of the other Th subsets such as IL-17 or IFN-γ, but in certain cases may co-express TNF-α and IL-2, suggesting functional distinctiveness from traditional Th1/Th17 cells 12, 30. Furthermore, evident cross-species differences are observed in MS-associated GM-CSF^+^ Th cells, with a Th1-biased phenotype in humans, while a Th17-like phenotype has been observed as predominant in mouse models 31, 32.

### 2.2 Conserved and species-specific regulation of GM-CSF^+^ Th differentiation

The differentiation of GM-CSF⁺ T helper cells follows a principle of “shared regulatory frameworks with disease- and species-specific modulation”. Core pro-differentiation pathways across contexts include the IL-12/STAT4, IL-23/RORγt, and IL-7/IL-2/STAT5 axes, which broadly support the generation and stabilization of GM-CSF-expressing subsets. Negative regulators such as TGF-β, IFN-γ, and IL-10 generally constrain GM-CSF production to prevent excessive inflammation 12, 13, 21. For example, IL-23 and IL-1β can induce GM-CSF production in helper T cells in a dose-dependent manner, whereas IFN-γ, IL-12, and TGF-β exert inhibitory effects 13, 14, 19, 20. The differentiation of GM-CSF⁺ T helper cells is governed by a conserved transcriptional network involving *STAT3/4/5*, *BHLHE40*, and *RUNX1/3*. *STAT5* mediates IL-2/IL-7 signals to promote the GM-CSF-producing phenotype, while *BHLHE40* directly binds the *CSF2* promoter to enhance transcription. *RUNX* factors contribute to T cell plasticity and effector programming. In autoimmune diseases such as MS and T1D, GM-CSF⁺ Th cells frequently co-express *T-bet* and *RORγt*, forming hybrid Th1/Th17-like subsets with potent pro-inflammatory capacity 20, 21, 27, 31-33.

Nevertheless, disease-specific heterogeneity has been repeatedly reported. In MS, human cases rely more on the IL-12/STAT4 pathway driving the Th1-like phenotype (T-bet^+^), while mouse models frequently exhibit a Th17-like phenotype predominantly driven by IL-23/RORγt 11, 14, 28. In contrast, GM-CSF^+^ Th cells in RA primarily differentiate into Th1-like phenotypes by IL-12/STAT4 or form a GM-CSF^+^IL-21^+^ subsets through CD147 activation 29. The BHLHE40-driven triple-positive phenotype (IFN-γ^+^IL-17A^+^GM-CSF^+^) in T1D and the IL-1β-driven transdifferentiation from Th17 to GM-CSF^+^ Th in IBD further suggest the strong impact of disease microenvironments on differentiation pathways 30, 33.

Difference between humans and mice involves not only preferences for core pathways but also regulatory factor specificity. For example, IL-2RA polymorphisms directly affect GM-CSF secretion in human MS, while the synergistic effect of IL-7 and IL-2 plays a more critical role in mice 34, 35. Additionally, the regulatory impact of Bhlhe40 on GM-CSF is significantly stronger in mouse models than in human subjects, suggesting possible species-specific compensatory mechanisms 26. Notably, although the IL-7/IL-2/STAT5 axis is evolutionarily conserved, IL-2 alone can drive GM-CSF secretion in humans, whereas in mice it is prerequisite a synergistic action of both IL-7 and IL-2. This difference is potentially related to species difference in IL-2R expression levels and STAT5 phosphorylation efficiency 21, 36.

### 2.3 Interplay Between heterogeneity and plasticity in GM-CSF^+^ Th cells

The pathogenic identity of GM-CSF-producing Th cells is defined not by a static phenotype, but by a dynamic interplay between their inherent heterogeneity and profound plasticity. This heterogeneity is evident at multiple molecular levels. In terms of cytokine profiles, beyond their hallmark GM-CSF secretion, these cells can co-express a range of effector molecules including TNF-α, IL-2, and IL-3. Depending on the microenvironment, they polarize into distinct functional subsets, such as IFN-γ-producing (Th1-like), IL-17A-producing (Th17-like), or highly pro-inflammatory hybrid subsets expressing both 14, 32. This functional diversity is underpinned by a flexible transcriptional network, where combinations of master regulators like T-bet and RORγt drive specific phenotypes in diseases like MS, while factors such as BHLHE40 can dictate unique triple-positive signatures in T1D 31, 33.

This spectrum of heterogeneity is not pre-determined but is actively and continuously shaped by the cells' remarkable plasticity. The core “rheostat” governing this plasticity is the dynamic balance of the IL-12 and IL-23 signaling axes. The IL-12/STAT4 pathway preferentially drives the Th1-like phenotype, prevalent in MS, while the IL-1β/IL-23/RORγt axis promotes the Th17-like phenotype often seen in other contexts 11, 37. Furthermore, the IL-7/IL-2/STAT5 axis plays a crucial role in maintaining this plastic potential, enabling cells to adopt different fates, such as the GM-CSF single-positive subset in GVHD 20, 26, 38. This foundational plasticity is then fine-tuned by disease-specific signals from the local microenvironment, for example, the IL-1β/NF-κB pathway in inflammatory bowel disease or CD147 signaling in rheumatoid arthritis, which ultimately sculpts the final pathogenic phenotype 29, 30.

While the foundational role of GM-CSF^+^ Th cells in autoimmunity is now established, several critical questions remain that will define the next phase of research.

First, moving beyond conserved pathways, how do disease-specific microenvironments, such as the gut microbiome in IBD, metabolic shifts in T1D, or the unique cytokine milieu of a tumor, precisely sculpt the phenotype and function of these cells? Understanding this context-dependent regulation is key to explaining clinical heterogeneity. Second, the significant discrepancies between human disease and murine models, particularly in the dominant Th1-like versus Th17-like polarization, represent a major translational hurdle. This necessitates not only large-scale clinical validation using patient samples but also the development of more sophisticated humanized models to bridge this gap. Finally, how do GM-CSF^+^ Th cells orchestrate the broader immune landscape? Elucidating their intricate crosstalk with other key players, such as regulatory T cells that restrain them and the diverse myeloid cell populations they activate, is essential to fully comprehend their net pathogenic impact.

In summary, GM-CSF^+^ Th cells function as highly adaptable “inflammatory amplifiers” within the autoimmune network. A deeper understanding of their heterogeneity and plasticity is the prerequisite for designing truly personalized therapies. The integration of multi-omics and single-cell analysis technologies will be instrumental in unraveling these complex dynamics, ultimately enabling us to target the right pathogenic subset, in the right patient, at the right time.

## 3. The Mechanistic Role of GM-CSF^+^ Th Cells in Pathogenesis of Various AIDs

### 3.1 Role of GM-CSF^+^ Th cells in multiple sclerosis

Multiple sclerosis (MS) is a chronic, immune-mediated demyelinating disease of the central nervous system (CNS), characterized by inflammation, myelin destruction, axonal loss, and neurodegeneration. Globally, MS affects an estimated 2.8 million individuals, with a prevalence of approximately 35.9 cases per 100,000 people, and shows striking geographical variation, being more common in high-latitude regions. Women are disproportionately affected, with a female-to-male ratio of approximately 3:1 39. The disease typically manifests in young adulthood (20-40 years of age), and its pathogenesis involves a complex interplay of genetic susceptibility, environmental triggers such as Epstein-Barr virus (EBV) infection and vitamin D deficiency, and aberrant immune regulation 40.

Historically, research into MS pathogenesis focused on the classic Th1 (IFN-γ^+^) and Th17 (IL-17^+^) lineages. However, their precise contributions were confounded by paradoxical observations. For instance, both genetic knockout of IFN-γ and antibody-mediated neutralization unexpectedly exacerbated experimental autoimmune encephalomyelitis (EAE), the primary mouse model for MS 8, 9. Clinically, some MS patients experienced disease worsening following IFN-γ therapy 41. Similarly, neither T-cell-specific IL-17A overexpression nor its complete deletion significantly altered susceptibility or the expansion of autoreactive T cells in murine EAE 42. These findings strongly suggested that the traditional Th1/Th17 paradigm was incomplete and that other critical, non-redundant mediators were essential for driving CNS autoimmunity.

The pivotal role of GM-CSF emerged from foundational studies. As early as 2001, McQualter et al. discovered that mice genetically deficient in GM-CSF (i.e., by genetically *Csf2*-knockout) were completely resistant to myelin oligodendrocyte glycoprotein (MOG)-induced EAE. Furthermore, when anti-GM-CSF monoclonal antibody is administered at the time of antigen challenge (i.e., during immunization with MOG₃₅₋₅₅ peptide), it can prevent clinical disease onset during the treatment period and for 10 days after discontinuation. However, when intervention occurs after disease establishment (i.e., after clinical symptoms occur and the scoring reaches two), it only alleviates symptoms, allowing mice to fully recover within 20 days after treatment 43. This foundational discovery implicated GM-CSF as a critical driver of disease initiation and an auxiliary factor in later stages. A subsequent landmark study in 2008 by Mark et al. directly linked this pathogenicity to T cells. They demonstrated that Th17-polarized cells required GM-CSF expression to become encephalitogenic, as adoptive transfer of GM-CSF^+^ Th17 cells was sufficient to induce EAE in naive recipients 44. These studies collectively established GM-CSF as a key, non-redundant pathogenic factor in EAE. The cytokine GM-CSF thus emerged as a non-redundant driver of CNS autoimmunity. Supporting this, McQualter et al. had earlier shown that *Csf2*⁻/⁻ mice were entirely resistant to MOG-induced EAE, and anti-GM-CSF antibodies administered during immunization blocked disease initiation but were less effective after disease onset. The pathogenic role of GM-CSF appears to be dynamic across the course of MS. It is considered a critical driver of early disease initiation, while transitioning to an auxiliary yet persistent function in sustaining inflammation during later stages. This is strongly supported by clinical cohort studies, which consistently demonstrate a significant correlation between elevated GM-CSF levels and disease activity 14, 35, 45-50.

Subsequent research confirmed that CD4^+^ T cells are the predominant source of pathogenic GM-CSF in both MS and EAE. In MS patients, the frequency of GM-CSF^+^ Th cells is elevated in peripheral blood and the CNS during active disease and decreases during remission following IFN-β therapy, correlating strongly with disease activity 35, 47, 51. This clinical observation was mechanistically validated in the FROG (fate-map and reporter of GM-CSF expression) mouse model, which enabled real-time tracking of GM-CSF-expressing cells. Komuczki et al. found that while various immune cells produced GM-CSF in peripheral lymph nodes post-immunization, CD4^+^ T cells were the dominant source within the CNS inflammatory milieu 20. This finding, supported by numerous studies 20, 23, 43, 52. solidifies that CNS pathology in EAE is primarily driven by GM-CSF produced by Th cells. Genetic evidence further reinforces this link, as an MS-associated polymorphism in the *IL2RA* gene (rs2104286) correlates with a higher proportion of GM-CSF^+^ Th cells 35, and IL-2 signaling is known to promote their differentiation.

Early studies described GM-CSF as a co-effector cytokine in Th1 and Th17 cells 31. However, subsequent work demonstrated that GM-CSF expression, not IL-17 or IFN-γ, was essential for encephalitogenicity. El-Behi et al. showed that IL-1β and IL-23 induced GM-CSF, rather than IL-17, to confer pathogenicity to Th17 cells 11. Similarly, Codarri et al. reported that RORγt promoted GM-CSF expression independently of IL-17 14.

A paradigm shift occurred in 2014 when Sheng et al. identified GM-CSF^+^ Th cells as a distinct, independent pathogenic lineage regulated by an IL-7-STAT5 axis, which they termed “ThGM”. In their model, conditional deletion of STAT5 in CD4^+^ T cells abrogated EAE by specifically preventing the development of ThGM cells, without affecting Th1, Th17, or Treg populations 21. Mice lacking STAT5 in CD4⁺ T cells failed to develop EAE despite intact Th1/Th17/Treg compartments, underscoring the indispensable role of GM-CSF⁺ Th cells.

Further studies identified GM-CSF⁺ Th subsets in MS patients that lacked IFN-γ and IL-17 but were dependent on IL-2/STAT5 signaling 13. IL-12 could induce IFN-γ expression in these cells, producing a GM-CSF⁺IFN-γ⁺ Th1-like phenotype 53. FROG mouse studies confirmed the presence of CNS-infiltrating ThGM cells in EAE, although many converted to IFN-γ⁺TNF-α⁺ “ex-GM-CSF⁺” cells over time 20, 23, 32. Spath et al. validated the pathogenicity of GM-CSF⁺ Th cells directly, as mice receiving GM-CSF-overexpressing CD4⁺ T cells developed spontaneous CNS inflammation, unlike those receiving IL-17-overexpressing cells 49. Research also found that the relatively EAE-resistant Albino Oxford (AO) rats showed restricted production, differentiation, and proliferative capacity of neural antigen-specific GM-CSF^+^ Th cells in their draining lymph nodes (dLNs), while EAE-susceptible Dark Agouti (DA) rats presented more pronounced GM-CSF^+^ Th cell-associated inflammatory responses in spinal cord (SC) tissues 54.

**Figure 3:**
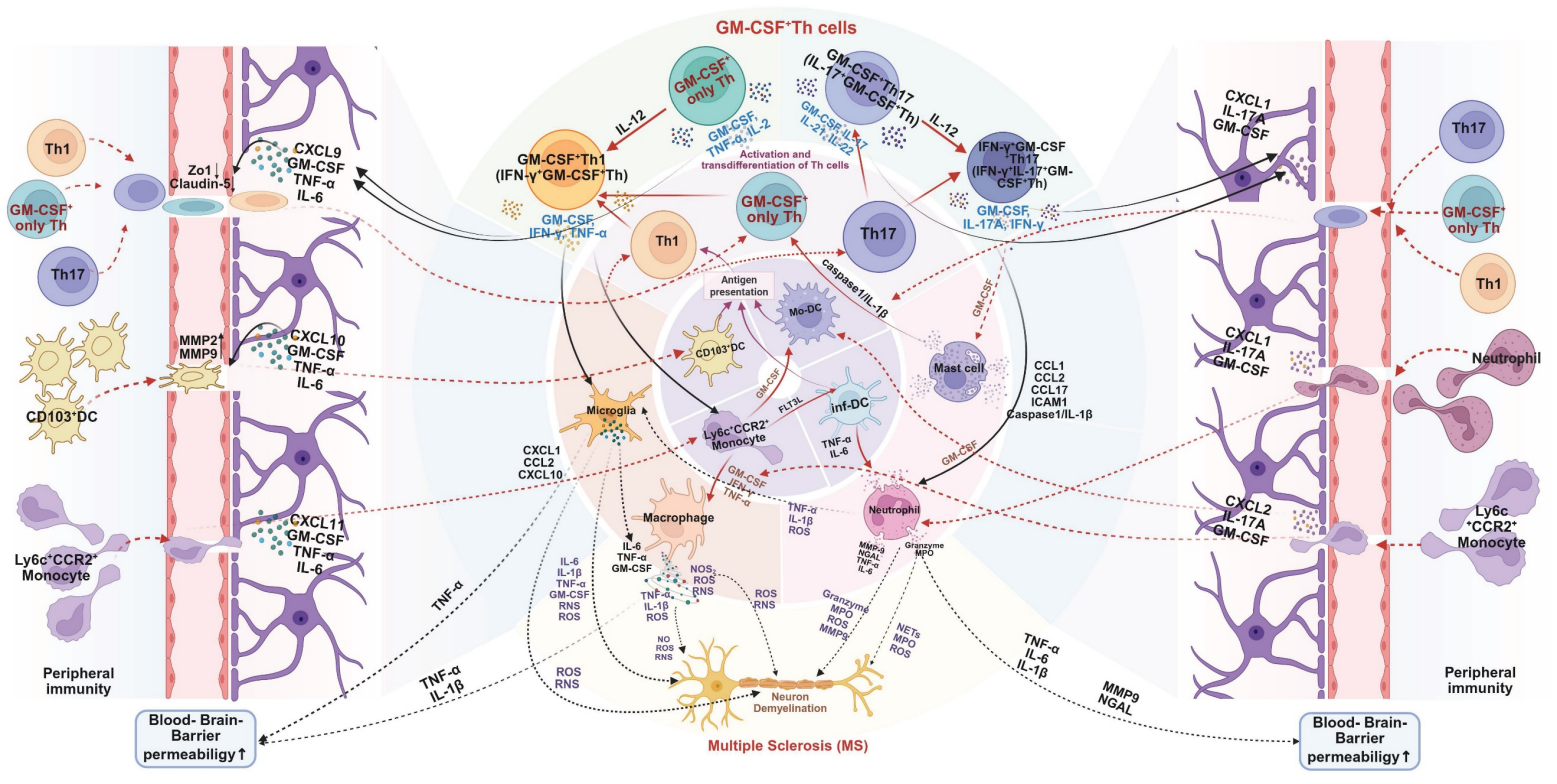
** Mechanistic role of GM-CSF^+^ Th cells in pathogenesis of multiple sclerosis (MS).** GM-CSF⁺ Th cells are pivotal in driving MS pathology. They initiate the disease process by increasing blood-brain barrier (BBB) permeability, allowing for the infiltration of peripheral immune cells into the central nervous system (CNS). Within the CNS, these Th cells orchestrate a self-amplifying neuroinflammatory cascade. They exhibit significant plasticity, such as IL-12-driven transdifferentiation into more pathogenic subtypes, and potently activate resident microglia and recruited myeloid cells (e.g., macrophages, neutrophils). This activated myeloid network releases a barrage of neurotoxic mediators, including cytokines, reactive oxygen species (ROS), and proteases, that directly cause the demyelination and neuronal damage characteristic of MS. Created in BioRender. Meng, X. (2025) https://BioRender.com/o6m32us.

These studies offer significant insights into the immunopathogenesis of MS and its murine model-EAE. Importantly, it is not merely that Th1 or Th17 cells act as passive carriers of GM-CSF-mediated pathogenicity. Rather, the acquisition of GM-CSF-producing capacity is a prerequisite for these T cell subsets to exert their full pathogenic potential in MS or EAE 55-57. In addition to Th1 and Th17 cells, independent CD4⁺ T cell subsets that produce high levels of GM-CSF, without expressing classic Th1 or Th17 markers, can also induce EAE, highlighting a broader spectrum of GM-CSF^+^ effector T cells. However, such findings from animal models should be interpreted with caution. Human MS pathology is multifaceted, and both Th1 and Th17 cells are widely distributed in MS lesions 58-60. Furthermore, the plasticity of CD4⁺ T cells allow for dynamic phenotype switching. For instance, IL-12 can induce IFN-γ expression in previously GM-CSF-only T cells, while Th1 and Th17 cells can acquire GM-CSF-producing ability under specific inflammatory conditions. This suggests that the actual pathogenic network *in vivo* involves a flexible and interconvertible pool of effector T cells, rather than fixed Th lineages 61. Despite the heterogeneity of contributing cell types, likely differing between individuals, GM-CSF secretion consistently emerges as a critical mediator of neuroinflammation. Thus, future research should prioritize elucidating the regulatory networks controlling GM-CSF production in CD4⁺ T cells. Special attention should be given to the distinctions between GM-CSF⁺ Th1, Th17, and non-classical effector subsets, and Treg, to identify novel therapeutic targets for preventing their differentiation or blocking GM-CSF secretion 62-64. Mechanistically, GM-CSF^+^ Th cells orchestrate CNS destruction through a multi-pronged assault (**Figure 3**). After differentiating in peripheral lymphoid organs, they cross the blood-brain barrier (BBB). Their secreted GM-CSF compromises BBB integrity by downregulating tight junction proteins, facilitating the infiltration of pro-inflammatory myeloid cells (e.g., CCR2^+^Ly6Chi monocytes) and other lymphocytes 64-66. Within the CNS, GM-CSF acts as a master regulator, promoting the differentiation of monocytes into inflammatory macrophages and activating microglia. These myeloid cells, in turn, release a barrage of destructive mediators, including pro-inflammatory cytokines (e.g., IL-1β, IL-6, TNF-α), reactive oxygen species (ROS), and reactive nitrogen species (RNS), which directly damage myelin and neurons 14, 43, 60, 67-72. GM-CSF also supports DC activation, autoantigen presentation, and Th cell proliferation in the CNS 73. This process creates a self-amplifying inflammatory loop, as GM-CSF also supports the survival and proliferation of the pathogenic Th cells themselves in an autocrine/paracrine manner, sustaining chronic inflammation 43, 67.

Clinically, MS pathology predominantly features macrophages with accompanying neutrophils 74. While pathophysiological heterogeneity is well-recognized across MS subtypes, such as Relapsing-Remitting (RRMS) and Progressive MS (PMS), a starkly different profile is observed in certain fulminant demyelinating disorders. For instance, Marburg's variant, historically misclassified as a rare MS subtype, is now predominantly considered a severe phenotype of MOG antibody-associated disease (MOGAD). It is pathologically characterized by extensive neutrophilic infiltration into lesions, leading to the release of matrix metalloproteinases (e.g., MMP-9) and ROS. This cascade results in widespread BBB disruption, fulminant demyelination, and an exceptionally rapid clinical progression driven by a hyperacute immune response 75, 76. EAE induced by GM-CSF⁺IFN-γ⁺ Th1-like cells is macrophage-predominant, resembling classical MS, while GM-CSF⁺IL-17⁺ Th17-like cells induce neutrophil-rich inflammation similar to Marburg MS 44, 77. This suggests that the specific sub-lineage of GM-CSF^+^ Th cells (Th1-like vs. Th17-like) can dictate the character of the resulting neuroinflammation. While GM-CSF^+^ Th1-like cells appear predominant in standard MOG-induced EAE models the balance between these subsets likely varies among human MS patients, contributing to clinical heterogeneity 20, 37.

A recent study reported that the frequency of GM-CSF⁺ Th17-like cells in C57BL/6 mice immunized with MOG positively correlates with EAE severity, suggesting that this specific Th subset may possess heightened encephalitogenic potential. These findings imply that the abundance of GM-CSF⁺ Th17-like cells could serve as a key determinant of disease intensity 11.

In clinical studies involving MS patients, the relative distribution of IFN-γ⁺GM-CSF⁺ versus IL-17⁺GM-CSF⁺ Th cells varies significantly across cohorts, indicating heterogeneity in the GM-CSF⁺ Th cell compartment among individuals. This observed diversity underscores the importance of conducting future clinical investigations using stratified analyses with adequately powered sample sizes to derive more definitive conclusions. Moreover, notable discrepancies exist between findings from EAE models and clinical observations in MS. For instance, while anti-IFN-γ monoclonal antibody treatment exacerbates disease in EAE models, administration of IFN-γ in some MS patients has been associated with clinical deterioration 11, 44. These contrasting outcomes suggest that IFN-γ may exert divergent effects in murine versus human systems. One potential explanation lies in the differential composition of Th cell subsets and the context-dependent influence of cytokine milieu, particularly IL-12 and IL-23, on Th cell plasticity and GM-CSF expression. In EAE, where Th17 cells may be more prominent in the CNS microenvironment, IFN-γ may act as a suppressor of GM-CSF secretion in GM-CSF⁺ Th17-like cells. Therefore, neutralizing IFN-γ in this context may relieve this suppression, enhance GM-CSF production, and exacerbate disease severity. Conversely, in MS patients whose CNS pathology is dominated by GM-CSF⁺ Th1-like cells, exogenous IFN-γ may amplify inflammatory cascades. These mechanistic nuances highlight the necessity for personalized treatment strategies that account for patient-specific Th subset profiles and cytokine dynamics, rather than applying uniform therapeutic approaches 78-80.

As illustrated in **Figure 3**, GM-CSF^+^ Th act as central orchestrators of MS and EAE pathogenesis through a combination of BBB disruption, activation of myeloid cells, induction of oxidative stress, and self-sustaining autocrine loops. Unlike the conventional Th1/Th17 paradigm, GM-CSF⁺ Th cells constitute a non-redundant and independently regulated effector subset. This distinct immunological identity opens new avenues for therapeutic intervention, including the use of anti-GM-CSF monoclonal antibodies and STAT5 inhibitors. Additionally, these findings reinforce the critical importance of timing in clinical intervention, particularly the stage of disease progression at which GM-CSF signaling is targeted. Nevertheless, the inconsistencies between EAE models and human MS, such as the opposing roles of IFN-γ, underscore the need for caution when extrapolating preclinical data to clinical practice. Tailored therapeutic strategies, guided by in-depth immunophenotyping and molecular profiling, may ultimately yield more effective and individualized treatment paradigms for MS.

Future research could prioritize several key areas. First, applying single-cell multi-omics technologies to dissect the dynamic heterogeneity and transcriptional or epigenetic regulatory networks of GM-CSF⁺ CD4⁺ T cells at high resolution. Second, refining therapeutic strategies, such as developing dual-targeted approaches that simultaneously inhibit GM-CSF and other pro-inflammatory cytokines (e.g., IL-17 or IFN-γ) implicated in MS pathogenesis. Third, improving animal models by constructing humanized mouse models that more faithfully replicate the phenotypic and functional characteristics of Th cell subsets observed in MS patients.

### 3.2 Role of GM-CSF^+^ Th cells in rheumatoid arthritis

Rheumatoid arthritis (RA) is a chronic autoimmune disease characterized by persistent synovitis, joint destruction, and systemic immune dysregulation, with a global prevalence of approximately 0.5-1%. Women are affected two to three times more often than men. The pathogenesis of RA involves a multifaceted interplay among genetic predisposition, environmental triggers, and aberrant immune responses 81. Although biologics and small-molecule inhibitors have substantially improved therapeutic outcomes, complete remission remains elusive, and long-term complications such as cardiovascular disease continue to threaten patient prognosis 82. Anti-citrullinated protein antibodies (ACPAs) can be detected 5-10 years before RA onset, but specific preventive targets and effective preventive measures are still lacking. Therefore, identifying key pathogenic factors is crucial for RA prevention. Recently, increasing attention has been directed toward GM-CSF⁺ Th cells and their secreted cytokine GM-CSF.

In a pioneering study using the collagen-induced arthritis (CIA) model, Ian K et al. demonstrated that GM-CSF-deficient (GM-CSF⁻/⁻) mice were resistant to arthritis, where heterozygous mice exhibited mild disease and wild-type mice developed severe arthritis, suggesting a dose-dependent contribution of GM-CSF to disease severity. Moreover, exogenous GM-CSF injection exacerbated joint damage, confirming GM-CSF's role as an endogenous pathogenic factor 83. In RA patients, elevated levels of GM-CSF are detected both in peripheral blood and synovial fluid, predominantly secreted by CD4⁺ Th cells. Peripheral GM-CSF⁺ Th cells exhibit minimal co-expression of other cytokines, whereas intra-articular GM-CSF⁺ Th cells often co-produce TNF-α and, in ~80% of cases, also express IFN-γ. IL-12 enhances the co-secretion of GM-CSF and IFN-γ, implicating the Th1 differentiation pathway 84. However, GM-CSF⁺IFN-γ⁺ Th cells in joints may not represent *bona fide* Th1 cells, but could instead derive from GM-CSF⁺ single-positive precursors in peripheral blood that acquire IFN-γ expression locally under IL-12 influence. Despite similar cytokine profiles, these cells may differ in origin and function, warranting further lineage-tracing investigation. *In vitro* studies corroborate that IL-12 can promote GM-CSF and IFN-γ production in human CD4^+^ T cells, while Th17-polarizing factors inhibit GM-CSF^+^ Th cell differentiation and GM-CSF secretion 13. Clinically, methotrexate (MTX) treatment leads to a significant reduction of GM-CSF⁺ Th cells in peripheral blood, correlating with disease activity and indicating their potential as dynamic biomarkers 85. Additionally, higher baseline proportions of GM-CSF⁺ Th cells are associated with superior therapeutic responses to TNF-α inhibitors, suggesting a predictive value for treatment stratification 86.

In the synovial fluid of RA patients, CD4⁺ T cells are the predominant source of GM-CSF, with significantly higher secretion than in healthy controls. GM-CSF^+^ Th cells promote monocyte differentiation into CD1c^+^ inflammatory dendritic cells (infDCs), which secrete pro-inflammatory cytokines such as IL-6 and TNF-α, thereby perpetuating inflammation through a feed-forward loop 87, 88. These infDCs contribute to T and B cell activation, maintain chronic inflammation, and promote joint damage by enhancing neutrophil gelatinase-associated lipocalin (NGAL), impairing chondrocyte function, and activating synoviocytes. Furthermore, monocyte-derived dendritic cells (MoDCs) differentiated by GM-CSF can process RA-associated autoantigens, the type II collagen (CII) and cartilage glycoprotein 39 (HCgp39), and present their immunodominant epitopes via MHC class II molecules, initiating autoreactive T cell responses 89-92. Taken together, GM-CSF⁺ Th cells drive multiple RA pathomechanisms, including inflammation, immune activation, and tissue damage, and are central players in sustained synovial pathology 43, 88. Their accumulation in inflamed joints offers mechanistic insight into the chronicity of RA and identifies them, along with GM-CSF itself, as potential therapeutic targets 93.

Despite encouraging preclinical results, clinical trials targeting GM-CSF have yielded mixed outcomes. While early-phase studies of GM-CSF-neutralizing antibodies (e.g., mavrilimumab) demonstrated reductions in inflammatory biomarkers, many patients failed to achieve primary or key secondary endpoints in phase III trials 94. This limited efficacy may reflect disease chronicity and refractoriness, as participants often had poor responses to prior synthetic and biologic disease-modifying antirheumatic drugs (DMARDs), including JAK inhibitors. Notably, mavrilimumab demonstrated greater efficacy in early RA, consistent with CIA model findings where early anti-GM-CSF intervention prevented disease onset, whereas late intervention only mitigated disease severity 95.

It is also essential to recognize fundamental interspecies differences in the biology of GM-CSF⁺ Th cells. In the SKG mouse model, GM-CSF augments macrophage production of IL-1β and IL-6 and promotes the expansion of both Th17 and GM-CSF⁺ Th cell populations 88. Notably, anti-GM-CSF therapy in this model yields greater suppression of arthritis severity compared to IL-17 blockade, underscoring a more central role for GM-CSF in disease progression 95. Mechanistically, murine Th17 cells are capable of producing GM-CSF upon IL-23 stimulation, and the GM-CSF⁺ Th17 phenotype is well-documented in experimental models of inflammatory arthritis. However, this immunological landscape diverges in human RA. Synovial tissues from RA patients typically exhibit low frequencies of IL-17-producing Th cells, and GM-CSF is predominantly produced by Th1-like CD4⁺ T cells co-expressing IFN-γ 96, 97. This Th1-skewed GM-CSF⁺ subset appears to be the dominant effector population in human RA, as supported by recent single-cell transcriptomic and mass cytometry studies 84, 88. Interestingly, recent investigations in SKG mice have identified IL-17A⁺GM-CSF⁺ Th cells enriched within inflamed joints and lymphoid tissues. Treatment with CCR4-targeted antagonists effectively suppressed their function, resulting in reduced synovitis and joint damage 97, 98. However, the actual abundance of these double-positive cells remains relatively low, and their IFN-γ expression status has not been systematically assessed, necessitating further investigation into their heterogeneity and relevance. The limited efficacy of anti-IL-17A monoclonal antibodies in RA clinical trials (e.g., secukinumab 99, brodalumab 100, CNTO6785 101) further underscores human-mouse differences, and suggests that IL-17-associated pathways, while prominent in murine arthritis, may play a subordinate role in human RA. These findings collectively highlight the need for cautious interpretation of Th cell-associated mechanisms across species and support a more targeted focus on Th1-like GM-CSF⁺ cells in human RA pathogenesis and therapy.

**Figure 4:**
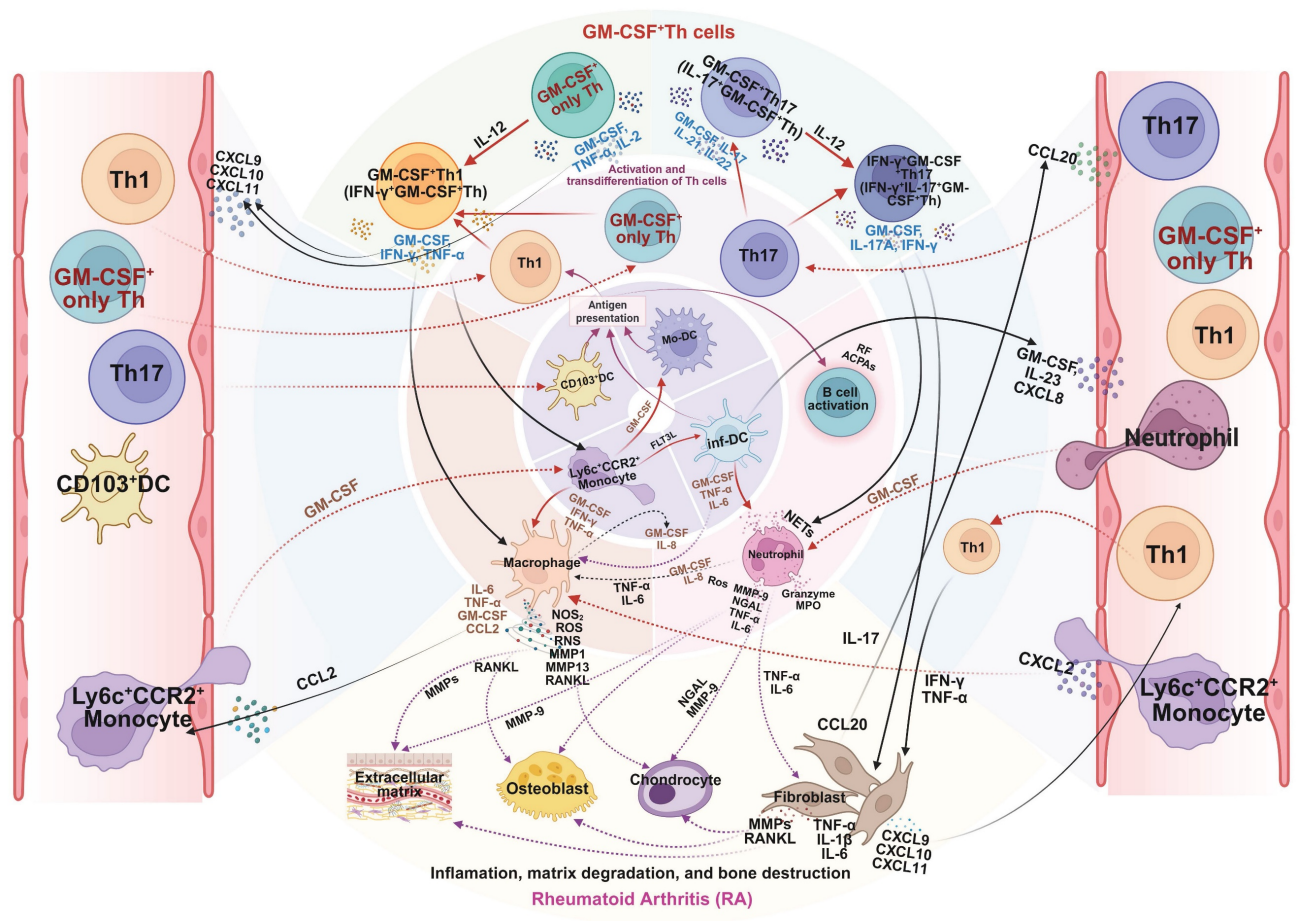
** Mechanistic role of GM-CSF^+^ Th cells in pathogenesis of rheumatoid arthritis (RA).** GM-CSF⁺ Th cells are key drivers of RA pathology. After infiltrating the synovial joint, they undergo local reactivation and differentiation, orchestrating a destructive inflammatory network. By secreting GM-CSF and other cytokines, they activate macrophages, neutrophils, and synovial fibroblasts, while also promoting autoantibody production by B cells. This cellular crosstalk unleashes a cascade of pathogenic mediators such as NOS, ROS, MMPs, and RANKL, leading directly to the cartilage degradation and bone erosion damage of RA. Created in BioRender. Meng, X. (2025) https://BioRender.com/o6m32us.

As summarized in **Figure 4**, GM-CSF^+^ Th cells function as a pivotal hub within the RA pathological network, amplifying inflammation, facilitating autoimmunity, and driving tissue destruction. Although current clinical strategies targeting GM-CSF are constrained by disease stage and patient heterogeneity, early intervention and combinatorial approaches merit further exploration. Future studies incorporating single-cell multi-omics and longitudinal tracking may refine patient stratification and optimize GM-CSF⁺ Th cell-centered therapeutic strategies.

### 3.3 Role of GM-CSF^+^ Th cells in miscellaneous autoimmune disorders

**Figure 5:**
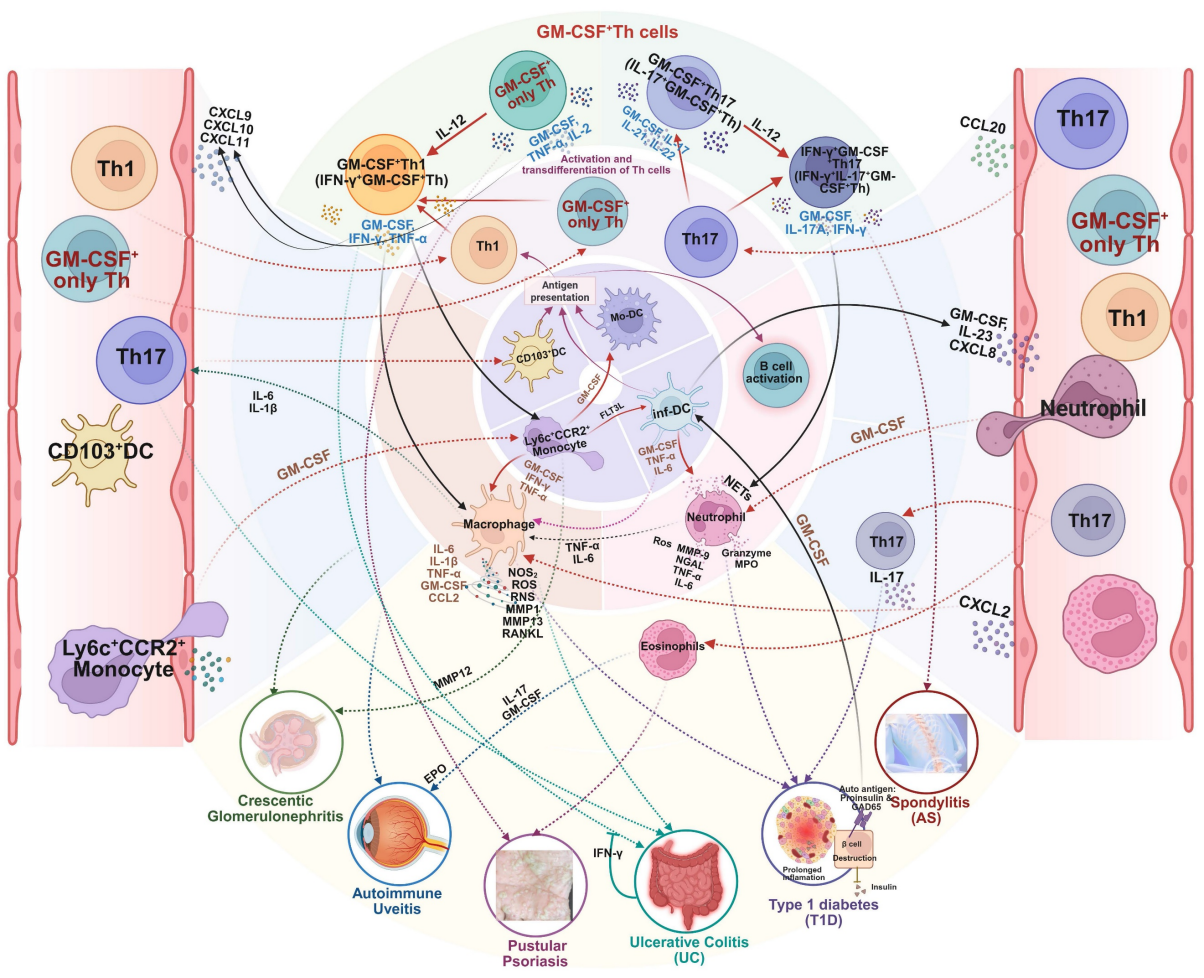
** The versatile mechanistic role of GM-CSF⁺ Th cells in the pathogenesis of miscellaneous autoimmune diseases.** GM-CSF⁺ Th cells orchestrate a central inflammatory hub that drives a wide range of autoimmune diseases. By activating a common set of myeloid effector cells, including macrophages, neutrophils, and eosinophils, this pathway can induce shared while tissue-specific pathologies. The specific clinical manifestations, be it the β-cell destruction in type 1 diabetes, joint inflammation in spondylitis, epithelial damage in pustular psoriasis and ulcerative colitis, or renal failure in crescentic glomerulonephritis, is determined by the target organ and the precise downstream inflammatory mediators that are engaged. Created in BioRender. Meng, X. (2025) https://BioRender.com/o6m32us.

The critical role of GM-CSF^+^ Th cells in the pathogenesis of a wide variety of other autoimmune diseases, such as type 1 diabetes, ulcerative colitis, and spondyloarthritis, are graphically summarized in **Figure 5**.

#### 3.3.1 Role of GM-CSF^+^ Th cells in type 1 diabetes

Type 1 diabetes (T1D) is an autoimmune disorder characterized by progressive and irreversible destruction of pancreatic β cells, ultimately resulting in insufficient insulin production 102, 103. CD4⁺ T cells are central to T1D pathogenesis, with early research identifying Th1 and Th17 subsets as key pathogenic drivers 104-107. However, these studies were largely constrained by a limited understanding of CD4⁺ T cell heterogeneity, possibly overlooking functionally important subsets. Recent advances have highlighted GM-CSF and GM-CSF⁺ Th cells as critical contributors to autoimmune pathology, including T1D 33, 106-109.

Emerging evidence suggests that GM-CSF⁺ Th cells play a central role in mediating β cell destruction in T1D. In patients with T1D, autoreactive CD4⁺ T cells targeting self-antigens such as proinsulin and glutamic acid decarboxylase 65 (GAD65) are elevated, with a significant increase in GM-CSF⁺ Th cells among them. These cells contribute to disease progression by exerting multiple pro-inflammatory effects via GM-CSF secretion. On one hand, GM-CSF promotes recruitment of immune cells—including macrophages and neutrophils—into pancreatic islets, enhancing local immune-mediated injury. On the other hand, GM-CSF facilitates Th17 polarization and IL-17 production, amplifying chronic inflammation and accelerating β cell destruction 110.

Moreover, GM-CSF⁺ Th cells often co-express IL-17A and IFN-γ, forming a pathogenic feed-forward loop wherein these pro-inflammatory cytokines mutually reinforce their expression. This loop intensifies local inflammation and directly contributes to β cell dysfunction and insulin insufficiency. Clinical studies show that T1D patients exhibit significantly higher peripheral levels of GM-CSF⁺ Th cells compared to healthy controls, correlating positively with plasma IL-17A, IFN-γ, and IL-2 levels 33, 110.

Findings from animal models and clinical studies further support the pathogenic role of GM-CSF⁺ Th cells in T1D. In clinical trials, T1D patients responding to alefacept therapy showed a marked reduction in circulating GM-CSF⁺ Th cells. Notably, baseline levels of these cells inversely correlated with post-treatment C-peptide retention, suggesting their potential as predictive biomarkers for therapeutic response 33, 108. Mechanistically, GM-CSF⁺ Th cells activate inflammatory dendritic cells (infDCs), enhancing their antigen-presenting capacity and breaking immune tolerance, ultimately leading to irreversible β cell destruction 110. In addition to producing GM-CSF, these Th cells release high levels of IL-17A, IFN-γ, and IL-2, establishing a pro-inflammatory islet microenvironment that perpetuates autoimmunity.

Although the regulatory mechanisms underlying GM-CSF⁺ Th cell differentiation remain incompletely understood, IL-23 has been implicated in promoting their expansion through specific signaling pathways. Ustekinumab, a monoclonal antibody targeting the p40 subunit shared by IL-12 and IL-23, has demonstrated efficacy in reducing GM-CSF⁺ Th cell numbers and alleviating β cell loss in T1D models 108. These findings indicate that ustekinumab may offer therapeutic benefit by modulating GM-CSF⁺ Th cell-mediated inflammation.

Further exploration of the transcriptional regulation and microenvironmental interactions of GM-CSF⁺ Th cells within pancreatic islets may uncover novel therapeutic targets. In particular, dual-targeted strategies that simultaneously address inflammatory signaling and immune cell migration hold promise for improving clinical outcomes in T1D. Despite ongoing challenges, GM-CSF⁺ Th cells are increasingly recognized as central amplifiers of inflammation and autoimmunity in T1D, offering a new immunological axis for therapeutic innovation.

#### 3.3.2 Role of GM-CSF^+^ Th cells in ulcerative colitis

Ulcerative colitis (UC) is a chronic inflammatory bowel disease (IBD) affecting millions worldwide, significantly reducing quality of life and increasing the risk of colorectal cancer. While its immune network is complex, recent research has identified GM-CSF-producing Th cells as key pathogenic drivers that amplify intestinal inflammation. This view is supported by foundational evidence from dextran sodium sulfate (DSS)-induced colitis models, where mice genetically deficient in GM-CSF (*Csf2*^-/-^) exhibit markedly reduced intestinal inflammation and less severe disease, establishing the necessity of GM-CSF in this pathogenic process 12.

Mechanistically, GM-CSF^+^ Th cells orchestrate intestinal damage through at least two interconnected pathways. First, they directly activate myeloid cells, such as macrophages, through the secretion of GM-CSF. These activated macrophages then release a storm of pro-inflammatory cytokines, including IL-1β and IL-6, which in turn drive the expansion of Th17 cells. This creates a potent, self-amplifying inflammatory loop: GM-CSF^+^ Th cells → GM-CSF → Macrophages → IL-1β → Th17 expansion. Second, adoptive transfer experiments have provided direct proof of their pathogenicity, confirming that GM-CSF+ Th cells are sufficient on their own to induce severe colitis, while their depletion or the neutralization of GM-CSF significantly alleviates disease 30.

Despite this strong evidence, the narrative is not without complexity. An early study from 2014 reported that conditional knockout of GM-CSF in CD4^+^ T cells did not prevent colitis induction, suggesting a less critical role 21. This discrepancy may be explained by the profound plasticity of T cells and the specific inflammatory context of different experimental models. More recent and detailed mechanistic work has clarified that in the gut, a unique IL-1β-driven pathway, rather than the canonical IL-23/RORγt axis, is responsible for inducing this pathogenic GM-CSF^+^ phenotype. This IL-1β signaling, acting via IRAK1/NF-κB, can also convert pre-existing Th17 cells into GM-CSF producers, explaining the frequent co-expression of IL-17 and GM-CSF observed in UC patients 30.

Perhaps the most unique aspect of UC immunopathology is the powerful regulatory role exerted by the intestinal epithelium itself. IFN-γ signaling in intestinal epithelial cells (IECs) has been found to be profoundly protective. In mice with an IEC-specific deletion of the IFN-γ receptor (IfngrΔIEC), GM-CSF^+^ Th cells accumulate uncontrollably in the gut 12. Mechanistically, IFN-γ signaling in IECs enhances their ability to present antigen and promotes the expression of CD39 on adjacent T cells. CD39 is an ecto-enzyme that degrades extracellular ATP, a potent danger signal. By clearing ATP, this pathway prevents the activation of the NLRP3 inflammasome in myeloid cells, thereby shutting down the production of IL-1β, the very cytokine required to drive the pathogenic GM-CSF^+^ Th cell program. When this IFN-γ-mediated epithelial “brake” is lost, the NLRP3/IL-1β pathway becomes hyperactive, fueling a vicious cycle of GM-CSF^+^ Th cell expansion and neutrophil infiltration 12, 30.

This intricate regulatory network highlights the therapeutic potential of targeting this axis. While anti-GM-CSF antibodies can alleviate inflammation, their efficacy may be limited unless combined with other immunomodulators. The unique dependency on the IL-1β/NLRP3 pathway suggests that inhibitors of this pathway could be particularly effective in UC. Furthermore, strategies that enhance the protective IFN-γ signaling within the intestinal epithelium could offer a novel approach to restoring immune homeostasis in the gut. Unraveling the distinct functional characteristics of tissue-resident versus circulating GM-CSF^+^ Th cells remains a key area for future investigation.

#### 3.3.3 Role of GM-CSF^+^ Th cells in less frequent autoimmune disorders

Beyond the major autoimmune diseases, the pathogenic footprint of GM-CSF-producing Th cells extends to a wide array of less common but severe disorders. While the specific clinical manifestations differ, a synthesis of these findings reveals a set of conserved, fundamental pathogenic principles that these cells employ to drive tissue damage.

A primary and universal mechanism is the recruitment and activation of myeloid cells. This principle is vividly illustrated across multiple organ systems. In the skin of patients with pustular psoriasis, a unique IL-23R^+^ subset of GM-CSF^+^ Th cells orchestrates the massive infiltration of neutrophils and monocytes, driving the formation of sterile pustules. Neutralizing GM-CSF in a preclinical model of this disease completely abrogates this myeloid influx and restores skin homeostasis 111. Similarly, in experimental autoimmune uveitis (EAU), GM-CSF^+^ Th cells specifically recruit eosinophils to the retina. These activated eosinophils then release toxic enzymes like eosinophil peroxidase (EPO), causing severe retinal damage and vision loss, an effect that is blocked by anti-GM-CSF antibodies 112.

A second core principle is the induction of destructive enzymatic pathways in target tissues. This is exemplified in crescentic glomerulonephritis (cGN), where GM-CSF+ Th1-like cells accumulate in the kidneys. The GM-CSF they produce activates infiltrating monocyte-derived cells, inducing them to secrete matrix metalloproteinase 12 (MMP12). This enzyme directly degrades the glomerular basement membrane, leading to irreversible kidney damage and functional decline. This discovery positions the GM-CSF/MMP12 axis as a key therapeutic target for preventing renal destruction in T-cell-driven nephritis 113.

Third, GM-CSF^+^ Th cells often act as initiators of a broader, self-sustaining inflammatory network. In spondyloarthritis (SpA), GM-CSF+ Th cells in the synovial fluid interact with dendritic cells, enhancing their antigen-presenting capacity and creating a persistent local immune response that drives chronic joint inflammation 114. A similar mechanism is observed in dry eye disease (DED), where GM-CSF^+^ Th17 cells in draining lymph nodes recruit and activate CD11b^+^ myeloid cells at the ocular surface, initiating a cycle of inflammation that can be disrupted by local administration of anti-GM-CSF antibodies 115.

Finally, the central role of these cells is underscored in the context of alloimmunity, such as in graft-versus-host disease (GVHD). Here, GM-CSF produced by donor-derived T cells is a critical factor that promotes myeloid cell production of IL-1β and ROS, driving multi-organ damage. The pathogenic importance of this axis is conserved across numerous other inflammatory conditions, including juvenile idiopathic arthritis, Behçet's disease, and endometriosis 34, 36, 116-122.

Moreover, as uncovered recently in an elegant piece of work by Gan and colleagues 123, GM-CSF-producing CD4^+^ T cells are integral to pro-inflammatory circuit that can sustain and amplify autoimmune responses through the signaling pathway involving GM-CSF, glucocorticoid-induced tumor necrosis factor receptor-related ligand (GITRL), and mTORC1, with the Sjögren's syndrome as a key example. In this autoimmune disease, the GM-CSF-GITRL-mTORC1 pathogenic loop has been shown to drive the differentiation and expansion of pathogenic Th17 cells secreting GM-CSF. Specifically, in this mechanism, GM-CSF stimulates monocytes to express GITRL, which then acts on CD4^+^ T cells through GITR to promote Th17 differentiation via mTORC1 pathway activation. The resulting pathogenic Th17 cells produce additional GM-CSF, creating a self-amplifying circuit that perpetuates inflammation. Flow cytometry analysis revealed that blocking GITRL significantly inhibited the expansion of Th17, Th17.1, and GM-CSF^+^ CD4^+^ T cells, while GM-CSF treatment dose-dependently enhanced GITRL expression on monocytes. Importantly, mechanistic studies confirmed that mTORC1 activation is essential for this process, as rapamycin treatment effectively inhibited GITRL-induced phosphorylation of S6 and STAT3 and suppressed Th17 expansion. The significance of this pathway was further validated *in vivo*, where administration of rAAV6-shRNA targeting GITRL alleviated disease progression in a NOD mouse model of Sjögren's Syndrome 123. This GITRL-mTORC1-GM-CSF feedback loop represents a key mechanism by which GM-CSF^+^ T cells not only function as downstream effectors but also actively shape pathogenic T cell responses through intercellular communication with monocytes. These findings highlight the complex regulatory networks involving GM-CSF^+^ T cells in autoimmune conditions and suggest potential therapeutic approaches targeting this pathway.

In conclusion, regardless of the specific disease context, be it the joint, skin, eye, or kidney, GM-CSF^+^ Th cells consistently function as central pathogenic nodes. They do so by executing a core playbook of disease-driving mechanisms: recruiting myeloid cells, inducing tissue-degrading enzymes, enhancing pathogenic Th cell response, and orchestrating self-sustaining inflammatory networks. A deeper understanding of these shared principles, combined with the study of disease-specific nuances, will be essential for developing effective, targeted therapies for this broad spectrum of devastating disorders.

## 4. Translational Applications Targeting GM-CSF^+^ Th Cells

GM-CSF⁺ Th cells represent a highly heterogeneous and plastic subset of helper T cells. Their indispensable role in the pathogenesis of multiple autoimmune diseases has been confirmed in both clinical and experimental settings. Defined by their dependence on STAT5/BHLHE40 signaling, co-expression of pro-inflammatory cytokines (e.g., IL-17A, IFN-γ), and close interaction with myeloid cells, GM-CSF⁺ Th cells offer promising targets for next-generation immunotherapies.

### 4.1 Cytokine- and receptor-based therapeutic strategies

Several cytokine pathways crucial for GM-CSF⁺ Th cell differentiation have been exploited therapeutically. For instance, IL-2/IL-2R signaling is known to promote GM-CSF⁺ Th differentiation through STAT5 phosphorylation. Clinical use of daclizumab, an anti-IL-2Rα monoclonal antibody, showed efficacy in reducing inflammatory activity in relapsing multiple sclerosis (MS) by limiting GM-CSF secretion 124, 125. However, its eventual market withdrawal due to severe immune-related adverse effects underscores the dual role of IL-2 signaling: promoting effector T cell activation while supporting Treg homeostasis 126. This dichotomy highlights the need for more selective downstream targets in the GM-CSF axis to avoid collateral immunosuppression.

Similarly, IL-7 and IL-15 have also been implicated in the differentiation of GM-CSF⁺ Th cells via STAT5 activation. *In vitro* studies demonstrate that both cytokines drive naïve CD4⁺ T cells toward the GM-CSF⁺ phenotype through STAT5 phosphorylation. Lusvertikimab (anti-IL-7R) has shown promising results in phase II trials for ulcerative colitis, while HuMax-IL15 (anti-IL-15) has demonstrated tolerability and efficacy in early-phase RA trials 127. These findings support the potential of IL-7 and IL-15 blockade to indirectly suppress GM-CSF⁺ Th cell expansion in autoimmune contexts. Ustekinumab, a monoclonal antibody targeting the p40 subunit shared by IL-12 and IL-23, has shown clinical efficacy in multiple autoimmune conditions—such as Crohn's disease 128, ulcerative colitis 129, plaque psoriasis (psoriasis vulgaris) 130, and type 1 diabetes 108. Although not directly targeting GM-CSF, blockade of IL-23 may reduce GM-CSF⁺ Th differentiation by disrupting upstream cytokine signals. However, its mechanism involves broader immunosuppressive effects, and its relevance to GM-CSF⁺ Th biology remains partially indirect.

### 4.2 Direct GM-CSF pathway inhibition

Anti-GM-CSF monoclonal antibodies represent a more direct strategy. MOR103 showed favorable safety and tolerability in phase 1b trials for MS 131. In RA, mavrilimumab (anti-GM-CSF receptor antibody) significantly reduced disease activity, particularly in early disease stages 132. Agents antagonizing GM-CSF or its receptor demonstrated certain promising efficacy performance and basically favorable safety profiles 94, 133-148, as summarized in **Table 2**. However, therapeutic outcomes may vary across patients, likely due to T cell heterogeneity and irreversible tissue damage in late-stage disease.

Notably, the novel anti-GM-CSF agent otilimab failed to meet primary and secondary endpoints in RA patients refractory to DMARDs or JAK inhibitors. Despite encouraging preclinical data, it showed no superiority over sarilumab, likely due to suboptimal patient selection and late-stage intervention 94. These findings support the importance of biomarker-based stratification and early therapeutic timing. Nevertheless, those mixed clinical outcomes observed with GM-CSF-targeting therapies, particularly the failure of otilimab in rheumatoid arthritis trials, need cautious interpretation and warrant deeper critical analysis. Several factors likely contribute to these inconsistent results. Patient heterogeneity represents a significant challenge, as inflammatory diseases encompass diverse endotypes with varying pathophysiological mechanisms and GM-CSF dependency. The timing of therapeutic intervention could be crucial, because many trials enrolled patients with established disease where irreversible tissue damage had already occurred, potentially diminishing efficacy. Additionally, single-target approaches may be insufficient for conditions with redundant inflammatory pathways. Biomarker-based stratification was basically absent in most trials, preventing identification of GM-CSF-dependent patient subgroups who might benefit most from the targeted therapy. Furthermore, dosing regimens may have been suboptimal, with insufficient target engagement at affected tissue sites. These considerations highlight the need for refined patient selection strategies using predictive biomarkers, earlier therapeutic intervention before tissue damage becomes irreversible, and potentially combination approaches targeting complementary pathways. Future trials should address these factors to optimize the therapeutic potential of GM-CSF pathway modulation in inflammatory conditions.

### 4.3 Inflammasome and IL-1 axis modulation

The NLRP3 inflammasome, which can promote GM-CSF production via IL-1β amplification, is another relevant target. MCC950, a selective NLRP3 inhibitor, has shown anti-inflammatory efficacy in multiple autoimmune disease models—including MS 149, 150, RA 151, and IBD 152, 153. However, its clinical application is hampered by pharmacokinetic and safety limitations. Moreover, disease-specific paradoxical effects, such as symptom exacerbation in Sjögren's syndrome 154, emphasize the complexity of inflammasome targeting.

Canakinumab, a monoclonal antibody against IL-1β, has demonstrated favorable safety and efficacy in autoimmune settings 155. Nevertheless, heterogeneity in trial populations limits its interpretability in disease-specific contexts, emphasizing the need for more refined cohort definitions in future RA studies.

### 4.4 Precision biomarkers and stratified therapies

Advances in mass cytometry and ELISA-based serum profiling enable dynamic tracking of GM-CSF⁺ Th cells or circulating GM-CSF levels. For example, a ≥50% reduction in GM-CSF⁺ Th cells correlates with improved joint swelling index in RA 29. Furthermore, IL2RA genetic polymorphisms (e.g., rs2104286) are associated with elevated GM-CSF⁺ Th frequencies 35, suggesting their utility in genotype-based treatment selection 27, 48, 156. These patients may particularly benefit from STAT5-targeted therapies or CRISPR-mediated interventions.

### 4.5 Targeting STAT5 and transcriptional networks

STAT5 is a critical regulator of GM-CSF⁺ Th cell fate. IL-2, IL-7, and IL-15 converge on STAT5 phosphorylation to drive GM-CSF⁺ Th differentiation. Although several STAT5 inhibitors (e.g., pimozide) have demonstrated efficacy in oncology 157, 158, off-target effects and structural homology with other STAT family members limit their specificity in autoimmunity. Furthermore, STAT5 plays essential roles in hematopoiesis and Treg maintenance, raising concerns about long-term safety.

To improve specificity, cell surface markers such as CD69, CXCR4, and CX3CR1 may be used to guide GM-CSF⁺ Th cell-selective targeting. In addition, transcription factors including BHLHE40, Rel, RUNX1, NLRP3, and T-bet have also been implicated in GM-CSF⁺ Th cell regulation, offering new molecular targets for therapy.

### 4.6 CRISPR-Cas9 gene editing

Recent advances in CRISPR-Cas9 gene editing provide precise tools for disrupting key transcriptional regulators. Lin et al. developed mouse models harboring Y694F and Y699F mutations in STAT5A/B, which abrogate phosphorylation-dependent GM-CSF⁺ Th cell differentiation 159. This method demonstrates greater specificity and durability than small-molecule inhibitors and allows for cell-type-restricted interventions. Similar approaches could target BHLHE40, NLRP3, and other GM-CSF-regulatory transcription factors. Nonetheless, CRISPR technology still faces hurdles, including off-target editing and delivery efficiency, which must be addressed for clinical translation.

Despite the immense promise of these strategies, significant translational hurdles remain before they can be widely implemented. The most formidable of these is the issue of cross-species differences. The markers used to identify GM-CSF^+^ Th cells, the specific cytokine cues that drive their differentiation, and even their dominant pathogenic phenotype can differ substantially between standard mouse models and human patients. For instance, while mouse EAE models often feature a prominent GM-CSF^+^ Th17-like response, the pathology in human MS is frequently dominated by a Th1-like counterpart. This chasm between preclinical models and human disease underscores the critical necessity of developing and validating therapeutic targets in more sophisticated systems, such as humanized mice or patient-derived organoid models.

Ultimately, effectively targeting these “inflammatory amplifiers” requires more than just better models; it demands a paradigm shift in our therapeutic philosophy. The future of treating autoimmunity lies in moving beyond broad, indiscriminate immunosuppression toward the goal of precision immune remodeling. This future will be built on a foundation of multi-dimensional strategies that leverage cutting-edge technologies like epigenetic editing to correct faulty gene programs, synthetic biology to create “smart” therapies (e.g., logic-gated CAR-T cells), and artificial intelligence to design patient-specific interventions based on complex multi-omics data. The ultimate goal is no longer simply to dampen the immune response, but to selectively erase pathogenic immune memory, restore self-tolerance, and rebuild a balanced and functional immune homeostasis, offering the potential for truly curative solutions for autoimmune diseases.

**Figure 6:**
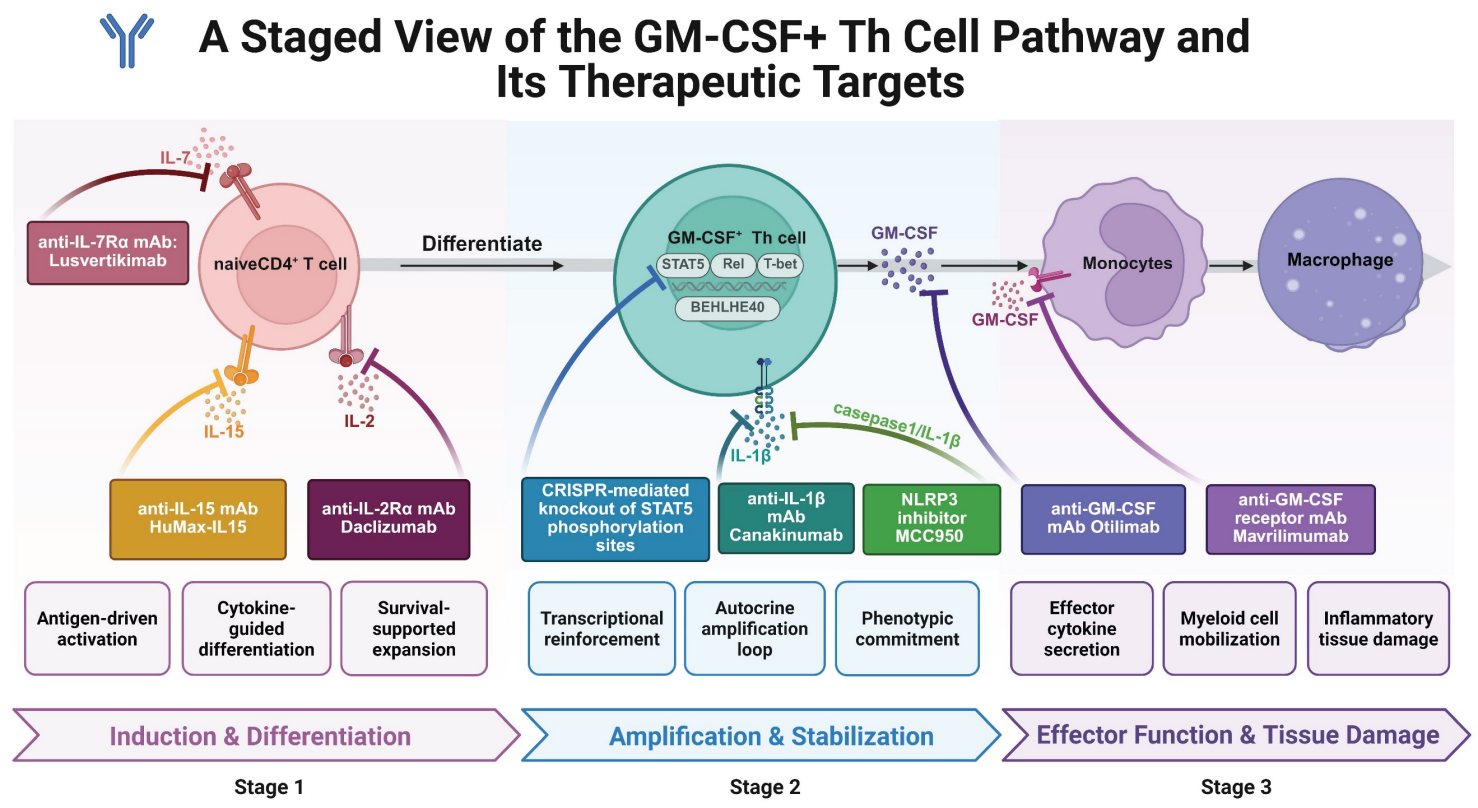
** Possible approaches for targeting GM-CSF^+^ Th cells.** This figure outlines a three-stage framework for therapeutically targeting the GM-CSF⁺ Th cell pathway, from initial development to final effector function. Stage 1 (Induction & Differentiation): This stage can be targeted by blocking the initial cytokine signals (e.g., IL-2, IL-7, IL-15) that drive the differentiation of naive T cells into the GM-CSF⁺ lineage. Stage 2 (Amplification & Stabilization): Intervention at this stage focuses on disrupting the transcriptional machinery and autocrine feedback loops (e.g., NLRP3/IL-1β) that reinforce and stabilize the pathogenic cell state. Stage 3 (Effector Function & Tissue Damage): The final stage offers therapeutic opportunities to neutralize the key effector molecule, GM-CSF (e.g., with Otilimab), or its receptor (e.g., with Mavrilimumab), thereby preventing the activation of myeloid cells and subsequent inflammatory tissue damage. Created in BioRender. Meng, X. (2025) https://BioRender.com/o6m32us.

A summary of these current and future therapeutic strategies is provided in **Table 3** and **Figure 6**.

## 5. Take-Home Message and Future Perspectives

GM-CSF⁺ Th cells constitute a highly heterogeneous and plastic subset of CD4⁺ helper T cells that act as “inflammatory amplifiers” in autoimmune diseases. Through the secretion of GM-CSF, these cells activate myeloid populations such as macrophages and dendritic cells, disrupt tissue barriers (e.g., the blood-brain barrier, pancreatic islets, or synovial membranes), induce oxidative stress, and engage in feed-forward loops alongside pro-inflammatory cytokines like IL-17 and IFN-γ, thereby driving disease progression. Across different disease contexts, GM-CSF⁺ Th cells exhibit disease-specific phenotypes and regulatory pathways. For instance, a Th1-like profile predominates in RA, whereas in T1D, a notable enrichment of IFN-γ⁺IL-17⁺GM-CSF⁺ triple-positive cells is observed. Their differentiation is governed by a network of signaling pathways, including IL-23/STAT3/RORγt, IL-7/STAT5, as well as epigenetic mechanisms. Despite the promising outcomes observed in preclinical models using GM-CSF-targeted therapies, including monoclonal antibodies, STAT5 inhibitors, and epigenetic modifiers, clinical translation remains hampered by several challenges. These include inter-patient heterogeneity in therapeutic efficacy, unclear windows for optimal intervention, and biological discrepancies between human disease and animal models.

Future research should aim to achieve mechanistic and translational breakthroughs in multiple dimensions. First, the integration of single-cell RNA sequencing (scRNA-seq), spatial transcriptomics, and epigenomic profiling will be instrumental in mapping the spatiotemporal evolution and plasticity of GM-CSF⁺ Th subsets across tissues and disease stages. Elucidating regulatory circuits, such as BHLHE40-RUNX1 interactions, may uncover lineage-defining checkpoints. Second, dissecting the metabolic programs of GM-CSF⁺ Th cells, including glycolysis and glutaminolysis, could enable the design of metabolic reprogramming strategies as adjunct therapies. Third, combining genetic risk variants (e.g., IL2RA, ZNF35), epigenetic biomarkers (e.g., GM-CSF promoter methylation), and immunophenotypic data (e.g., from multi-omic profiling) will facilitate the development of predictive biomarker systems for therapeutic stratification.

Next-generation therapeutic designs, such as logic-gated CAR-Tregs or switchable STAT5 inhibitors, may achieve spatiotemporal control of GM-CSF⁺ Th cell activity while minimizing systemic immunosuppression. Combinatorial approaches targeting GM-CSF alongside key inflammatory pathways (e.g., IL-1β, IL-23, or CXCL10) may overcome the challenge of cytokine redundancy; for instance, combining anti-GM-CSF monoclonal antibodies with JAK inhibitors could yield synergistic effects. Multifunctional nanomedicines that simultaneously suppress inflammation and promote tissue regeneration (e.g., β-cell recovery or remyelination) may offer a comprehensive therapeutic paradigm.

Advances in translational modeling, including organoid-immune cell co-culture systems and humanized mouse models, can more accurately replicate human disease microenvironments and bridge cross-species gaps in drug efficacy. Finally, longitudinal cohort studies in high-risk populations (e.g., preclinical T1D or RA) may help define the critical threshold of GM-CSF⁺ Th cell expansion, guiding early and precisely timed interventions.

## 6. Concluding Remarks

Research on GM-CSF^+^ Th cells is catalyzing a paradigm shift of autoimmune disease management from broad-spectrum immunosuppression to precision-guided immune remodeling. In the coming decades, the integration of multi-omics technologies, gene editing, and artificial intelligence is expected to drive the development of targeted therapies capable of halting, or potentially reversing, autoimmune pathology by modulating this pathogenic subset. However, several key challenges must be addressed to facilitate successful clinical translation. These include elucidating the mechanistic basis of phenotypic heterogeneity and functional plasticity among GM-CSF⁺ Th subpopulations, establishing real-time monitoring systems to track their dynamics in vivo, and ensuring therapeutic safety across diverse patient populations. Ultimately, it is only through sustained interdisciplinary collaboration, bridging medical immunology, bioinformatics, bioengineering, and clinical rheumatology, that curative strategies can be realized to effectively confront the complexity of autoimmune diseases.

## Supplementary Material

Supplementary table.

## Figures and Tables

**Table 1:** Subtypes of GM-CSF+ Th cells, their cellular origin, molecular characteristics, driving and suppressing factors, down-stream recruited inflammatory cells, and associated autoimmune diseases.

GM-CSF+ Th subtypes	Origin	Molecular characteristics		Drivers		Suppressors		Recruited infCells†	Associated Ads*	References
		Human	Mouse	Human	Mouse	Human	Mouse			
GM-CSF^+^ only Th cells	naïve CD4^+^ T cells	Markers: GM-CSF^+^, IL-2^+^, TNF-α^+^, CCR4^+^, CCR10^+^, CXCR4^+^, CD154^+^; IL-4^-^, IL-17A^-^, IFN-γ^-^, CCR6^-^, CCR7^‑^, CXCR3^-^, CXCR5^-^, CD25^‑^, CD27^-^, CD137^-^; IL-3^+/-^, CCL20^+/-^; Transcriptional factors: ZNF35^+^, SP2^+^, TEF^+^, TWIST1^+^, MSC^+^, TRERF1^+^, PPARG^+^; GATA3^-^, RORγt^-^, T-bet^-^	Markers: GM-CSF^+^, IL-2^+^, IL-3^+^, IL-15Rβ, IL-23R^+^, TNF-α^+^, CCL5^+^, CXCL9^+^, CCR1^+^, CCR4^+^, CCR9^+^, CCR10^+^, CX3CR1^+^, CD11A^+^, CD11C^+^, CD49D^+^, CD54^+^, CD69^+^, CD95^+^, CD147^+^, α4β7^+^; IL-17A^-^, IL-4^-^, IFN-γ^-^, CCR7^-^, CCR8^-^, CD45^-^, PD-1^-^, LAG3^-^; Transcriptional factors: Bhlhe40^+^, TWIST1^+^, MSC^+^, TRERF1^+^, PPARG^+^, NFATC1^+^, AP-1^+^, RUNX1^+^, BATF^+^, ST2^+^; T-bet^-^, RORγt^-^, GATA3^-^;	IL-2, IL-7, IL-15, IL-6, IL-12, STAT5, ZNF35, SP2, TEF;	IL-2, IL-7, IL-23, CD5L, IL-1β, IL-1R1, GM-CSF, STAT4, STAT5, BATF, NLRP3, Rel;	TGF-β, IL-6, IL-12, IL-1β, IL-23, IFN-γ, IL-13, IL-21; 5A12, MTX, IFN-β, STAT3, RORγt, Treg, DMF;	IL-4, IL-12, IFN-γ, Treg	Eosinophils, ly6C^+^CCR2^+^MCs, Neutrophils, DCs	MS, EAE, EAU, RA, T1D, IBD, SpA, Psoriasis	12, 14, 15, 19, 20, 21, 22, 23, 27, 84, 28, 30, 32, 35, 34, 38, 42, 50, 53, 86, 88, 109, 114, 112, 119, 120
GM-CSF^+^ IFN-γ^+^ Th cells (GM-CSF^+^ Th1-like)	GM-CSF^+^ only Th cells	Markers: GM-CSF^+^, IFN-γ^+^, TNF-α^+^, IL-2^+^, IL-2RA^+^, CXCR4^+^, CD2^+^, CD154^+^, CD161^+^; IL-17A^-^, CCR7^-^, CD25^-^, CD27^-^, CD137^-^; IL-3^+/-^, CCL20^+/-^, Transcriptional factors: T-bet^+^, ZNF35^+^, SP2^+^, TEF^+^, RUNX3^+^, RORC2^+^, BHLHE40^+^, TNFRSF9^+^, RORγt^-^;	Markers: GM-CSF^+^, IFN-γ^+^, TNF-α^+^, CCR1^+^, CX3CR1^+^, CD11a^+^, CD49d^+^, CD54^+^; IL-17A^-^; Transcriptional factors: T-bet^+^, RUNX3^+^; RORγt^-^;	IL-12, T-bet;	IL-7, IL-12, RUNX3, T-bet, Bhlhe40;	IL-23, IL-1β, IL-6, TGF-β, RORγt;	IL-6, IL-12, IL-21, IL-23, IFN-γ, TGF-β;	ly6C^+^CCR2^+^MCs, lymphocytes, neutrophils, macrophages, DCs	MS, EAE, MS; RA, JIA, T1D, IBD, SpA, GN;	11, 12, 13, 20, 21, 22, 23, 26, 27, 84, 31, 32, 33, 35, 37, 38, 42, 47, 50, 53, 71, 86, 88, 98, 109, 110, 113, 114, 118, 122
	Th1	Markers: GM-CSF^+^, IFN-γ^+^, TNF-α^+^, CCR4^+^, CCR6^+^, CCR10^+^, CXCR3^+^, IL-17A^-^, Transcriptional factors: T-bet^+^, RORγt^-^,	Markers: GM-CSF^+^, IFN-γ^+^, TNF-α^+^, CXCR6^+^; IL-17A^-^; Transcriptional factors: T-bet^+^, Bhlhe40^+^; RORγt^-^;	IL-12, STAT4;	IL-7, IL-12, IFN-γ, T-bet, Bhelhe40;	IL-1β, IL-6, IL-23, IL-21, IL-27, TGF-β, RORγt, STAT3	IL-10, IL-23;			
GM-CSF^+^ IL-17^+^ Th cells	Th17	Markers: GM-CSF^+^, TNF-α^+^, IL-17A^+^, IFN-γ^-^; Transcriptional factors: RORγt^+^, T-bet^-^;	Markers: GM-CSF^+^, IL-17A^+^, IL-17F^+^, IL-21^+^, IL-22^+^, TNF-α^+^, CCL20^+^, CCR4^+^, GPR65^+^; IFN-γ^-^, Transcriptional factors: Bhlhe40^+^, RORγt^+^, BATF^+^, T-bet^-^;	IL-12, GRP65, IL-15, IL-23;	IL-12, IL-7, IL-15, IL-1β, IL-23, IL-17, RORγt, Bhelhe40;	IFN-γ, Ustekinumab	IL-10, IL-12, IL-27, IFN-γ, TGF-β;	neutrophils, ly6C^+^CCR2^+^MCs, DCs;	EAE, MS, MS Marburg, CIA, T1D, IBD, SpA;	11, 12, 13, 14, 21, 22, 23, 26, 28, 30, 31, 32, 33, 35, 37, 38, 42, 71, 97, 98, 108, 109, 110, 113, 115, 119
GM-CSF^+^ IL-17^+^IFN-γ^+^ Th cells	GM-CSF^+^ IL-17^+^ Th cells	Markers: GM-CSF^+^, IL-2^+^, IFN-γ^+^, TNF-α^+^, IL-17A^+^; Transcriptional factors, RORγt+, T-bet^+^, BHLHE40^+^	Markers: GM-CSF^+^, IFN-γ^+^, IL-17A^+^, TNF-α^+^; Transcriptional factors: RORγt^+^, T-bet^+^, Bhlhe40^+^	IL-12, BHELHE40	IL-7, IFN-γ, IRF1, Bhlhe40	Ustekinumab		neutrophils, ly6C^+^CCR2^+^MCs, DCs	EAE, MS, MS Marburg, CIA, T1D, IBD, SpA	12, 21, 26, 33, 38, 42, 108, 110

†, inflammatory cells recruited by GM-CSF^+^ Th cells by subtypes. DCs, dendritic cells; MCs, monocytes; *, Autoimmune diseases associated with GM-CSF^+^ Th cells by subtypes. CIA, collagen-induced arthritis; EAE, experimental autoimmune encephalomyelitis; EAU, experimental autoimmune uveoretinitis; GN, glomerulonephritis; IBD, inflammatory bowel disease; JIA, juvenile idiopathic arthritis; RA, rheumatoid arthritis; SpA, spondyloarthritis; T1D, type 1 diabetes;

**Table 2:** Tabular summary of clinical trials of anti-GM-CSF agents in autoimmune disorders

Target	Molecule	Drug Type	Indication	Phase	Status	ClinicalTrials.gov identifier	Efficacy outcomes	Safety profiles	Reference
GM-CSFR	Mavrilimumab KPL-301 (aka, CAM-3001)	Monoclonal antibody	RA	I	Completed	NCT00771420	A significant reduction in disease activity was observed in patients with high baseline disease activity	Safety was favorable with most adverse events being mild to moderate, with no SAEs or DLTs.	133
			RA	II	Completed	NCT01050998	Mavrilimumab significantly increased the proportion of patients achieving a reduction in DAS28-CRP score at Week 12 (55.7% vs. 34.7%, *P* = 0.003). The 100 mg dose group demonstrated significant effects across multiple efficacy endpoints.	Mild to moderate adverse events such as infections and hypersensitivity reactions were observed.	134
			RA	IIa	Completed	NCT01050998	Mavrilimumab demonstrated significant efficacy across multiple dose groups, especially in the 30 mg and 100 mg dose groups. Improvements in DAS28-CRP and HAQ-DI scores were notable.	Most adverse events were mild to moderate. Overall tolerability was favorable.	135
			RA	IIb	Completed	NCT01706926	Mavrilimumab significantly reduced disease activity in RA patients with clinically meaningful responses observed as early as in Week 1. ACR20 response rates were significantly higher in all dose groups compared to placebo.	No treatment-related safety signals were identified, demonstrating good tolerability.	136
			RA	IIb	Completed	NCT01706926	Mavrilimumab significantly reduced inflammatory markers and improved RA symptoms. The efficacy was associated with the inhibition of myeloid and T-cell signaling pathways.	Overall tolerability was favorable. No serious adverse events were reported, demonstrating a favorable safety profile.	137
			RA	IIb	Completed	NCT01715896	Mavrilimumab demonstrated clinical efficacy in patients with refractory RA.	51.4% of patients in the mavrilimumab treatment group reported treatment-related adverse events. No deaths or specific safety signals were observed.	138
			RA	IIb	Completed	NCT01712399	Mavrilimumab showed sustained efficacy in long-term treatment, significantly improving disease status. Biomarker analyses indicated effective control of inflammatory response.	Long-term treatment was well-tolerated, with no increase in treatment-related adverse events. Common adverse events were mild to moderate and transient in nature.	139
GM-CSF	GSK 3196165 Otilimab (aka, MOR103)	Monoclonal antibody	RA	Ib/IIa	Completed	NCT01023256	Clinical efficacy included reductions in disease activity scores and joint counts with higher EULAR response rates.	MOR103 was well-tolerated in patients with moderate RA. No serious adverse events were reported.	140
			RA	IIa	Completed	NCT02799472	Some positive results were observed in target engagement and biomarkers, but significant improvements in synovitis were not achieved.	Otilimab was well-tolerated. No serious adverse events or deaths were reported.	141
			RA	IIb	Completed	NCT02504671	Although otilimab combined with methotrexate did not meet the primary endpoint of DAS28-CRP remission, improvements in disease activity scores were observed. Pain and physical function reported by patients improved significantly.	Otilimab was well-tolerated. No deaths or clinically significant pulmonary events were reported.	94
			RA	III	Completed	NCT04134728	Otilimab showed no superiority in efficacy compared to placebo.	Safety was acceptable.	142
			RA	III	Completed	NCT03980483, NCT03970837	In two trials, otilimab increased the proportion of patients achieving low disease activity per CDAI and reduced HAQ-DI scores compared to placebo.	The safety profile of otilimab was comparable to placebo and tofacitinib. No new safety concerns were identified.	143
			RA	III	Completed	NCT04333147	Otilimab demonstrated some efficacy, with stable response rates for patients achieving low disease activity per CDAI.	In long-term studies (<2.5 years) of otilimab for RA, safety was favorable. The incidence of adverse events was low.	144
	Namilumab (previously MT203)	Monoclonal antibody	RA	Ib	Completed	NCT01317797	A reduction in disease activity scores and improvement in joint symptoms were observed.	Namilumab was well-tolerated in patients with mild to moderate RA. Safety was comparable to placebo.	145
			PP	II	Completed	NCT02129777	Namilumab showed no significant efficacy in reducing PASI scores compared to placebo.	Safety was favorable, with no serious adverse events or significant drug-related safety issues identified.	146
			RA	II	Completed	NCT02379091	The primary endpoint was achieved at Week 12, showing a clear dose-response effect.	Tolerability was favorable. No serious infections were reported.	147
			axSpA	II	Completed	NCT03622658	Namilumab showed no efficacy in patients with active axSpA compared to placebo.	Tolerability was favorable.	148

RA, rheumatoid arthritis; PP, plaque psoriasis; axSpA, axial spondyloarthritis; DAS28-CRP, disease activity Score in 28 joints using C-reactive protein; ACR, American college of rheumatology; HAQ-DI, health assessment questionnaire disability Index; EULAR, European alliance of associations for rheumatology; CADI, clinical disease activity index; PASI, psoriasis area and severity index.

**Table 3:** Potential strategies for autoimmune disease management through targeting GM-CSF^+^ Th cells.

Potential Targets	Drug types	Agents	Disease* & development stage	Adverse effects & limitations	Possible strategies for improvement
GM-CSF	anti-GM-CSF mAb	Otilimab	RA, phase III^94^	drug resistance, immunosuppression, lung injury (proteinosis)	precise patient stratification and biomarker - guided treatment; optimization of dosing regimens; enhancement of safety monitoring and management; combination therapy strategies synergizing with other immunosuppressive drugs; improvement of patient compliance; conducting additional real - world studies.
GM-CSF receptors	anti-GM-CSF receptor mAb	Mavrilimumab	RA, phase IIb^132^	limited response rate, immunosuppression	
IL-2 receptors	anti-IL-2Rα mAb	Daclizumab	MS^124^], 125, approved (2016), withdrawn (2018) 126	severe inflammatory brain disease, hepatic injury, drug-induced rash, allergic reactions	
IL-7 receptors	anti-IL-7Rα mAb	Lusvertikimab	UC, phase II (CoTikiS)	immunosuppression; impaired immune tolerance, formation of anti-drug antibodies	
IL-15	anti-IL-15 mAb	HuMax-IL15	RA, phase I/II^127^	mild to moderate infections, NK cell function impairment, possible tumorigenicity,inadequate efficacy	precision patient selection, e.g. overexpression of GM-CSF+ Th1/Th17 cells and IL-15 both; integrating disease staging criteria into treatment decisions, e.g focusing on early-stage disease;
STAT5	small-molecule targeted inhibitors	Pimozide (repositioning)	inhibiting STAT5 phosphorylation in cells^157^, ^158^	mechanistic complexity, lack of human study, off-target effect;	targeted delivery of STAT5 inhibitor to GM-CSF-expressing Th cells with STAT5 overexpression;
IL-12 & IL-23 (P40)	anti-(IL-12 and IL-23) P40 mAb	Ustekinumab	clinical trial: Crohn's Disease^128^, UC^128^, Psoriasis^130^, T1D^108^	limited clinical data, immunosuppression, individual variability, potential impacts on adolescent development	evaluating IL-12/IL-23 p40 as a biomarker for stratifying patients
NLRP3	NLRP3 inflammasome inhibitor	MCC950	preclinical studies in animal models: MS^149^, ^150^, RA^151^, UC^152^, [153	limited clinical data, hepatotoxicity, immunosuppression, off-target effects, tissue-specific variability	developing oral prodrugs and nanoparticle-delivered long-acting sustained-release formulations to improve bioavailability; stratifying patients using NLRP3 genetic markers; designing bispecific antibodies targeting NLRP3 and IL-1β to mitigate immunosuppression-related risks; engineering reversible inhibitors to preserve host defense during infections
IL-1β	anti-IL-1β mAb	Canakinumab	RA, phaseI/II^155^	immunosuppression, individual variability in efficacy, high cost	screening patient populations responsive to IL-1β inhibition; developing long-acting sustained-release and oral formulations.
STAT5/T-bet/BHLHE40/Rel	Conditional gene editing or Small-molecule inhibitor	CRISPR-mediated knockout of STAT5 phosphorylation sites	mouse model with tyrosine-to-phenylalanine mutations (Y694F and Y699F) in STAT5A and STAT5B^159^	o-target effects, Editing efficiency, cell or tissue specificity, potential immune response	optimizing sgRNA design targeting GM-CSF^+^ Th cells, using Cas9 variants, combining with other editing techniques, improving delivery systems, Inducible gene knockout, multiplex gene editing, functional validation and compensation mechanism studies

* MS, multiple sclerosis; RA, rheumatoid arthritis; T1D, type 1 diabetes; UC, ulcerative colitis.
