# Supplementary material for: GM-CSF+ Th: a central player in autoimmunity
Source: Theranostics. 2025 Sep 12;15(18):9944–68. doi: 10.7150/thno.121766 (PMC12486991; doi:10.7150/thno.121766)
Supplement: Supplementary file 1 — Supplementary table. [file thnov15p9944s1.pdf]

Table S1. Literature associated with GM-CSF+ Th in autoimmunity

Disease	Terminology reported	Marker	Differentiation (human)		Differentiation (animal model)		Mechanism regulating differentiation	PMID	Year	Title
			Promote	Suppress	Promote	Suppress				
MS(mouse)	(1)GM-CSF^{+}Th1 cells; (2)GM-CSF^{+}Th17 cells	(1)GM-CSF+Th1 cells: GM-CSF+IFN-γ^{+}TNF-α^{+}L-17^{-}T-bet^{+}； (2)GM-CSF+Th17 cells: GM-CSF^{+}IL-17^{+}TNF-α^{+}IFN-γ$^{+/-}$RORγt^{+}			(1)GM-CSF+Th1 cells: IL-12; T-bet (2)GM-CSF+Th17 cells: IL-17; IL-23; RORγt; STAT3	GM-CSF+Th17 cells: IFN-γ	(1)IL-23/STAT3/RORγt/Th17/GM-CSF(Pro); （2）IL17/IL17R/NF-κB or MAPK/Th17/GM-CSF(Pro); (3)RORγt/Th17/GM-CSF(Pro); （4）IL-12/T-bet/Th1/GM-CSF(Pro); （5）IFN-γ/Th17/GM-CSF(Inh);	PMID: 18573909	2008	IL-12- and IL-23-modulated T cells induce distinct types of EAE based on histology, CNS chemokine profile, and response to cytokine Inhion
MS(human)	GM-CSF-only T cells; GM-CSF-producing Th1-like cells (dominant)	(1)GM-CSF-only T cells: GM-CSF^{+}IL-4^{-}IFN-γ^{-}IL-17^{-}T-bet^{-}RORγt^{-}GATA-3^{-} Foxp3^{-}CCR3^{+}CCR6^{+}CCR10^{+}; (2)GM-CSF-producing Th1-like cells: GM-CSF^{+}IL-4^{-}IFN-γ^{+}IL-17^{-}T-bet^{+}RORγt^{-} GATA-3^{-}Foxp3^{-} CCR3^{+}CCR6^{+}CCR10^{+}	IL-12； IL-2; STAT5	IL-6; IL-23; IL-21; STAT3；TGF-β；RORγt			(1)IL-12/STAT4/T-bet/Th1/GM-CSF(Pro); (2)IL-2/STAT5/naive CD4+T /GM-CSF(Pro); (3) IL-1β + IL-6 or TGF-β + IL-6/ Th/GM-CSF(Inh); (4) IL-23/ROR-γt/Th/GM-CSF(Inh);	PMID: 24944195	2014	IL-17 and GM-CSF Expression Are Antagonistically Regulated by Human T Helper Cells
MS(mouse)	GM-CSF-secreting helper T cells	GM-CSF^{+}RORγt^{+}IL-17$^{+/-}$			IL-23; RORγt	IL-12; IFN-γ; IL-27	(1)IL-23/RORγt/Th17/GM-CSF (Pro); (2)IL-12 or IL-27 or IFNγt/RORγt/Th17/GM-CSF (Inh)	PMID: 21516112	2011	RORγt drives production of the cytokine GM-CSF in helper T cells, which is essential for the effector phase of autoimmune neuroinflammation”
MS(mouse)	Lymph node-derived donor encephalitogenic CD4^{+} T cells	GM-CSF^{high} T-bet^{high}			IL-12; IL-23; IL-1β; T-bet;		（1）IL-12/STAT4/T-bet/Th/GM-CSF(pro); （2)IL-23+IL-1β/JAK-STAT/T-bet/Th7/GM-CSF(pro)	PMID: 21702922	2011	Lymph node-derived donor encephalitogenic CD4+ T cells in C57BL/6 mice adoptive transfer experimental autoimmune encephalomyelitis highly express GM-CSF and T-bet”

MS(mouse)	GM-CSF^{+}Th17 cells；IL-17A^{-} GM-CSF^{+}Th cells；GM-CSF^{+}Th1 cells	(1)GM-CSF+Th17cells: GM-CSF^{+} IL-17A^{+}RORγthighT-bethigh; (2)IL-17A-GM-CSF+Th cells: GM-CSF^{+}IL-17A-RORγt-T-bet-; (3)GM-CSF+Th1 cells: GM-CSF^{+}IFN-γ+T-bethigh			IL-23; IL-1β; T-bet ; RORγt	TGF-β	(1)IL-23/IL-23R/STAT3/Th17/GM-CSF (Pro); (2)IL-1β/NF-κB/Th17/GM-CSF (Inh); (3)IL-1β-T-bet /Th1/GM-CSF (Pro); (4) RORγt /Th17/GM-CSF (Pro); (5)TGF-β/Th17 /GM-CSF (Inh)	PMID: 21516111	2011	The encephalitogenicity of T(H)17 cells is dependent on IL-1- and IL-23-induced production of the cytokine GM-CSF
MS(mouse)	GM-CSF-producing TH1 cells; GM-CSF-producing TH17 cells	GM-CSF-producing T_H1 cells(GM-CSF^{+}IFN-γ^{+}*Bhlhe40* $^{+}$T-bet^{+}); GM-CSF-producing T_H17 cells(GM-CSF^{+}IL-17A^{+}Bhlhe40^{+} RORγt^{+})			*Bhlhe40; IL-23*	IL-10	(1)*Bhlhe40* /Th1 or Th17/GM-CSF(Pro); (2) IL-23/Th17/GM-CSF(Pro); (3)IL-10/Th1 or Th17/GM-CSF (Inh)；	PMID: 24699451	2014	Bhlhe40 controls cytokine production by T cells and is essential
MS(human)	GM-CSF-secreting Th cells ; GM-CSF+Th cells	GM-CSF^{+} IL-2Rα^{+}STAT5^{+}IFN-γ$^{+/-}$IL-17A^{+few}CCL5^{+}CXCL9^{+}	IL-2; IL-2Rα; IL-15; IL-7; STAT5	TGF-β1; TGF-β3;IL-6;IL-12;Treg			（1）IL-2 or IL-15 or IL-7/IL-2Rα /STAT5/naïve CD4+ Th/GM-CSF(Pro); (2)TGF-β1 or TGF-β3 or IL-6 or IL-12/ naïve CD4+ Th/GM-CSF(Inh); (3)Treg/IL-2/naïve CD4+ Th/GM-CSF(Inh)	PMID: 25278028	2014	Multiple sclerosis-associated IL2RA polymorphism controls GM-CSF production in human TH cells
MS(mouse)	GM-CSF-producing T helper cells, T_H-GM	GM-CSF^{+}IL-3^{+}STAT5^{+}T-bet^{low}RORγt^{low}			IL-7	IL-12、IFN-γ、TGF-β、IL-6; IL-23	(1)IL-7//IL-7R/STAT5/naïve CD4^{+} Th/GM-CSF(Pro); (2)IL-6 or TGF-β or IL-12 or IL-23 or IFN-γ/naïve CD4+ Th/GM-CSF(Inh)	PMID: 25412660	2014	STAT5 programs a distinct subset of GM-CSF-producing T helper cells that is essential for autoimmune neuroinflammation

MS(mouse)	GM-CSF-producing T helper cells; GM-CSF^{+}Th1 cells；GM-CSF^{+}Th17 cells; GM-CSF^{+} Th1/Th17	GM-CSF+IFN-γ$^{+/-}$IL-17$^{+/-}$			IL-7; IL-23		IL-7/IL-7Rα/Th0 or Th1 or Th17/GM-CSF(Pro); IL-23/ Th17/GM-CSF(Pro)	PMID:26223651	2015	IL-7/IL-7R signaling differentially affects effector CD4+ T cell subsets involved in experimental autoimmune encephalomyelitis1
MS(mouse)	IL-12-polarized Th1 cells; IL-23-polarized Th17 cells	(1)IL-12-polarized Th1 cells: GM-CSF^{+}IFN-γ^{+}IL-17^{-}Tbx21^{+}; (2)IL-12-polarized Th17 cells: GM-CSF^{+}IL-17^{+}RORγt^{+};			(1)IL-12-polarized Th1 cells: IL-12; IFN-γ; (2)IL-23-polarized Th17 cells: IL-23	IL-23-polarized Th17 cells: IL-12, IFN-γ; IL-12-polarized Th1 cells: IL-23	(1)IL-12 or IL-12+IFN-γ /Th1/GM-CSF(Pro); (2)IL-23 /Th17/GM-CSF(Pro); (3)IL-12 or IFN-γ /Th17/GM-CSF(Inh); (4)IL-23 /Th1/GM-CSF(Inh)	PMID: 26220255	2015	IL-12-polarized Th1 cells produce GM-CSF and induce EAE independent of IL-23
MS(human)	GM-CSF^{+}IFN-γ^{+}T cell; GM-CSF only T cell	(1)GM-CSF^{+}IFN-γ^{+}T cell: GM-CSF+IFN-γ+T-bet+; (2)GM-CSF only T cell: GM-CSF+IFN-γ- IL-17A-IL-4-T-bet- RORγt-GATA3-CXCR3-CCR4-CCR6-		IFN-β			IFN-β /GM-CSF only T cell/GM-CSF(Inh)	PMID: 25917097	2015	Expression of GM-CSF in T cells is increased in multiple sclerosis and suppressed by IFN-β therapy
MS(human)	CD4^{+}CD28^{-} T	GM-CSF^{+}IL-17^{-}IFN-γ$^{+/-}$CD28^{-} CD11a^{+}CD49d^{+}CD54^{+}CCR1^{+}CX3CR1^{+}	IL-15				IL15/IL-15Rβ/CD4^{+}CD28^{-} T cells/GM-CSF	PMID: 25617471	2015	IL-15 Amplifies the Pathogenic Properties of CD4^{+}CD28^{-} T Cells in Multiple Sclerosis

MS(mouse)	GM-CSF-producing Th cells	GM-CSF^{+}IL-17$^{+/-}$			IL-1β; IL-12; IL-23		(1)Mast cell/caspase-1/IL-1β /Th17/GM-CSF; (2)IL-23/ Th17/GM-CSF(Pro); (3)IL-12/ Th1/GM-CSF(Pro)	PMID: 27396526	2016	Meningeal mast cell-T cell crosstalk regulates T cell encephalitogenicity
MS(mouse)	GM-CSF-producing Th cells; GM-CSF+Th cells	GM-CSF^{+}IFN-γ $^{+}$ TNF^{+}CXCR6^{+} IL-4^{-} IL-10^{-} IL-2^{-} *RUNX1* ^{high}*RUNX2* ^{high}*Erg* ^{high}*ETV4* ^{high} *FEV* ^{high}*BATF* ^{high} *FOS* ^{high}*JUN* ^{high}； GM-CSF+only(few)； GM-CSF^{+}IFN-γ $^{+}$IL-17A^{+}（few）;GM-CSF^{+}IL-17A^{+}（few）；			IL-2; IL-23; IL-1β; IL-7	IL-6; IL-21;TGF-β; IL-12; IFN-γ	(1)IL-23 or IL-1β or IL-2 or IL-7 /naïve CD4+T cells/GM-CSF (Pro); (2)IL-6 or IL-21 or TGF-β or IL-12 or IFN-γ /Th/GM-CSF (Inh)	PMID: 31079916	2019	Fate-Mapping of GM-CSF Expression Identifies a Discrete Subset of Inflammation-Driving T Helper Cells Regulated by Cytokines IL-23 and IL-1β
MS(human)	GM-CSF+Th cells	GM-CSF^{+}IL-13^{+}						PMID:31237799	2020	T cells producing GM-CSF and IL-13 are enriched in the cerebrospinal fluid of relapsing MS patients
MS(human)	GM-CSF producing Th cells; GM-CSF^{+}Th cell	GM-CSF^{+}TNF-α^{+}IL-2^{+}IFN-γ$^{+(1/3)/-}$IL-3(few)$^{+/-}$IL-4$^{+(few)/-}$IL-6$^{+(few)/-}$IL-13$^{+/-}$ IL-21$^{+/-}$ IL-17A^{-}IL-6$^{+(few)/-}$IL-22^{-}IL-10^{-} CXCR4^{+}CCR4$^{+/-}$CCR6$^{+/-}$VLA4$^{+/-}$ T-bet^{-}RORγt^{-}(GM-CSF producing Th cells are heterogeneous.)	IL-2; IL-23; IL-1β	Dimethyl fumarate (DMF)			(1)IL-2/STAT5/naïve CD4+Th/GM-CSF;(pro); (2)IL-23 or IL-1β /Th17/GM-CSF;(pro); (3)DMF/GM-CSF^{+}CXCR4^{+} Th /GM-CSF(Inh)	PMID: 31332391	2020	GM-CSF and CXCR4 define a T helper cell signature in multiple sclerosis

MS(human)	T_HGM cells ; GM-CSF$^+$$T_H$1 cells(Major pathogenic subset) ; GM-CSF+T_H1/17 cells	(1)T_HGM: GM-CSF+TNF--α$^+$IL-2$^+$IL-3$^+$CCL20$^+$CD45RA$^-$IFN-γ$^-$IL-17A$^-$IL-4$^-$T-bet$^-$IL-22$^-$RORγt$^-$GATA3$^-$; (2)GM-CSF$^+$$T_H$1: GM-CSF$^+$IFN-γ$^+$TNF-α$^+$IL-2$^+$IL-3$^+$CXCR3$^+$CD45RO$^+$IL-17A$^-$IL-4$^-$T-bet$^+$RORγt$^-$GATA3$^-$; (3)GM-CSF+TH1/17 cells： GM-CSF$^+$IL-17$^+$IFN-γ$^+$TNF-α$^+$IL-2$^+$IL-3$^+$CXCR3$^+$CD45RO$^+$)	IL-6; IL-7; IL-12		IL-1β; IL-2; IL-12	IL-23-polarized Th17 cells: IL-12, IFN-γ; IL-12-polarized Th1 cells: IL-23	(1)IL-7/STAT5/naïve CD4+T/ThGM（human）(Pro); (2)IL-6/naïve CD4+T/ThGM（human）(Pro); (3) IL-1β/NF-κB/Th17 or naiveCD4+T/GM-CSF（mouse）(Pro); (3) IL-23/STAT3/RORr-γt/Th17/GM-CSF（mouse）(Pro);(4) IL-12/STAT4/T-bet/Th1/GM-CSF（mouse）(Pro)	PMID: 33097590	2020	A distinct GM-CSF+ T helper cell subset requires T-bet to adopt a TH1 phenotype and Pro neuroinflammation
MS(human and mouse)	GM-CSF pruducing Th1 or Th17	GM-CSF$^+$TNF+IFN-γ$^+$/IL-17$^{+/-}$(human)； GM-CSF$^+$TNF$^+$IFN-γ$^{+/-}$IL-17$^{+/-}$IL-15Rβ$^+$(mouse)；	IL-15; IL-2		IL-15/IL-15Rα		(1)IL-15 or IL-2/STAT5/naïve CD4+T or Th17/GM-CSF;	PMID: 33323466	2021	Interleukin-15 enhances proinflammatory T-cell responses in patients with MS and EAE
MS(human)	GM-CSF-producing CD4 T cell; GM-CSF-only-producing Th cells	GM-CSF+TNF-α+/-IL-2+/-IFN-γ+/-IL-17+(few)/-IL-4+(few)/-CXCL8+CXCL5+CCR6+CD3E+IL-2Rα+ZNF35highSP2highTEFhigh	ZNF35； SP2； TEF				ZNF35 or SP2 orTEF /naiveCD4+T cells/GM-CSF(pro)	PMID:34285924	2021	Gene Regulatory Network of Human GM-CSF-Secreting T Helper Cells
MS(mouse)	GM-CSF$^+$IL-17$^-$CD4$^+$ T cell; GM-CSF+Th17 cell	(1)GM-CSF$^+$IL-17-CD4$^+$ T cell: GM-CSF$^+$IL-17$^-$; (2)GM-CSF$^+$Th17 cell:GM-CSF$^+$IL-17$^+$			IL-23(p19)+CD5L		(1)IL-23(p19)+CD5L/STAT5/Th17 or naïve CD4+T or ThGM /GM-CSF(Pro)	PMID: 33664371	2021	IL-23p19 and CD5 antigen-like form a possible novel heterodimeric cytokine and contribute to experimental autoimmune encephalomyelitis development

MS(human and mouse)	ThGM cells （Human and mouse);IFN-γ^{+}GM-CSF^{+} CD4^{+}T cells (Enhanced encephalitogenicity, mouse)	(1)ThGM cells: GM-CSF+TNF-α+IL-2+IL-3+IFN-γ-CCR4 + CCR10+ CXCR3 -CXCR5 -CD25 -TWIST1+MSC+TRERF1 +PPARG+IFN-γlow IL-17Alow IL-4low IL-5lowIL-9low GATA3lowCXCR6 high CD103high CD27low; (2)IFN-γ+T-bet+RUNX3+ ThGM cells			IL-1β	IFN-γ,IL-4	IL-1β+anti-IFN-γ+anti-IL-4/naïve CD4+T cells/GM-CSF(Pro)	PMID: 35860266	2022	Transcription Factor RUNX3 Mediates Plasticity of ThGM Cells Toward Th1 Phenotype
RA(human)	GM-CSF-producing CD4+ T cells; GM-CSF+ T cells	GM-CSF^{+}CD45RO $^{+}$	IL-12; IL-15;	IL-1β;TGF-β ; IL-23;IL-6;IL-13；IFN-γ			(1)IL-12 or IL-15 /STAT5/naïve CD4+T/GM-CSF(Pro);(2)IL-1β or IL-6 or TGF-β or IL-23 or IL-13/IL-2/CD2+CD3+CD28/naïve CD4+T/GM-CSF(Inh);（3）IFN-γ /Th/GM-CSF(Inh)	PMID: 25923217	2015	Synovial CD4+ T-cell-derived GM-CSF supports the differentiation of an inflammatory dendritic cell population in rheumatoid arthritis
RA(human)	GM-CSF-producing T cells(GM-CSF+T cells)	GM-CSF^{+}CD45RO $^{+}$TNF-α$^{+/-}$IL-1β$^{+/-}$ IIL-6$^{+/-}$L-17$^{+(few)/-}$						PMID: 28488248	2017	Response to Treatment with TNFα Inhors in Rheumatoid Arthritis Is Associated with High Levels of GM-CSF and GM-CSF+ T Lymphocytes
RA(human)	(1)GM-CSF-only T cells; (2)GM-CSF-producing Th1-like cells(dominant); (3)GM-CSF-producing Th17-like cells(few)	(1)GM-CSF-only T cells：GM-CSF^{+}TNF-α$^{+/-}$IFN-γ^{-}IL-17^{-}; (2)GM-CSF-producing Th1-like cells: GM-CSF^{+}TNF-α$^{+/-}$IFN-γ^{+}IL-17^{-}IL-21$^{+/-}$; (3)GM-CSF-producing Th17-like cells: GM-CSF^{+}TNF-α$^{+/-}$IFN-γ^{-}IL-17^{+};						PMID: 28955490	2017	Th1 is the predominant helper T cell subset that produces GM-CSF in the joint of rheumatoid arthritis

RA(human)	CD147+GM-CSF+Th	CD147+GM-CSF+		5A12 (anti-CD147)			CD147 - 5A12 Fab /ZAP70 - LAT - ERK/Th/GM-CSF	PMID: 29563614	2019	A critical epitope in CD147 facilitates memory CD4+ T-cell hyper-activation in rheumatoid arthritis
RA(mouse)	GM-CSF-expressing Th17 cells	GM-CSF^{+}IL-17A^{+}CCR4^{+}CD44^{+}IL-17F^{+}IL-21^{+}IL-22^{+}				IL-23	IL-23/STAT3/Th17/GM-CSF(Pro)	PMID: 35997787	2022	CCR4 plays a pivotal role in Th17 cell recruitment and expansion in a mouse model of rheumatoid arthritis
RA(human)	GM-CSF^{+}CD4^{+} T-cells	GM-CSF^{+}TNF-α^{+}IFNγ^{+}IL-17$^{+(few)/-}$ /CCR7^{-}CD45RA^{-}		Methotrexate (MTX)				PMID: 38372731	2024	Rheumatoid arthritis synovial fluid shows enrichment of T-cells producing GMCSF which are polyfunctional for TNFα and IFNγ
JIA(human)	GM-CSF^{+}T cells ;	GM-CSF+CD161+RORC2+FN-γ +(80.1%)/-IL-17+（10%）/-					IL-12/Th17/GM-CSF+IFN-γ(Pro)	PMID: 24692225	2014	T Cell Expression of Granulocyte–Macrophage Colony-Stimulating Factor in Juvenile Arthritis Is Contingent Upon Th17 Plasticity

Interstitial Lung Disease in SKG Mice	GM-CSF-producing CD4+ T cells	GM-CSF^{+}IL-17^{+}						PMID:28805019	2017	CD11b+Gr-1dim Tolerogenic Dendritic Cell–Like Cells Are Expanded in Interstitial Lung Disease in SKG Mice
T1D（human）	GM-CSF^{+} CD4^{+} T cells	（1）GM-CSF^{+}IL-2^{+}TNF-α^{+} IFNγ^{-} IL17A^{-}IL-21^{-}IL-22^{-}CD45RA^{-} CD154^{high}FOXP3^{low}CD27^{low}CD25^{low}CCR7^{low}CD137 ^{low}；（2）GM-CSF^{+}IFNγ^{+}IL-17A$^{+/-}$IL-17A+/-IL-21$^{+/-}$ IL-22$^{+/-}$IL-2^{+}TNF-α^{+} CD45RA^{-} CD154^{high}FOXP3^{low}CD27^{low}CD25^{low}CCR7^{low}CD137 ^{low}						PMID: 29229565	2018	GM-CSF producing autoreactive CD4^{+} T cells in type 1 diabetes
T1D(human)	IAR CD4^{+} T cells	GM-CSF^{+}TNF-α^{+}IFN-γ^{+}IL-2^{+}IL-17A^{+}BHLHE40^{+}TNFRSF9^{high}IL2RA^{high}CD2^{high}IL-2RA^{high}TNFRSF9^{high}IL-7R^{low}	BHLHE40				BHLHE40/CD4^{+}T cell/GM-CSF(Pro)	PMID:37751304	2023	Islet-autoreactive CD4+ T cells are linked with response to alefacept in type 1 diabetes
T1D(human)	IL2+GM-CSF+ Th17.1 cells	GM-CSF^{+}IL-17A^{+}IFNγ^{+} IL-2^{+}BHLHE40^{+}	(IL-12; IL-23)p40	Ustekinumab			Ustekinumab/(IL-12, IL-23)p40/Th17.1/GM-CSF(Inh)	PMID: 39079992	2024	Ustekinumab for type 1 diabetes in adolescents: a multicenter, double-blind, randomized phase 2 trial

IBD(mouse)	T_H-GM-CSF cells	(1))GM-CSF^{+}IFN-γ^{-}IL-17^{-}RORγt^{low}AHR^{low} IRF4^{low}			IL-1β;IL-23;IL-1R1; STAT4		(1)IL-1β/IL-1R/IRAK1/naïve CD4+T cels/ NF-κB /P65/GM-CSF(Pro);(2)IL-1β/STAT4/naïve CD4+T cels/GM-CSF(Pro);(3)IL-1β /Th17/GM-CSF(Pro);	PMID: 31080014	2019	Interleukin-1β-induced IRAK1 ubiquitination is required for T(H)-GM-CSF cell differentiation in T cell-mediated inflammation.
IBD(mouse)	GM-CSF+ CD4+T cells	GM-CSF^{+}IL-17A$^{+/-}$IFN-γ$^{+/-}$			NLRP3, IL-1β	IFN-γ, IRF1	（1) IL-1β/NLRP3/naive CD4 + T cell/GM-CSF（Pro); (2)IFN-γ/IRF1/ GM-CSF+Th/GM-CSFl(Inh)	PMID: 37993709	2023	Epithelial IFNγ signalling and compartmentalized antigen presentation orchestrate gut immunity
SpA(human)	GM-CSF^{+} CD4 T cells; IL-17+GM-CSF+ Th cells; IFN-γ^{+}GM-SF^{+}Th cells	(1)GM-CSF^{+} CD4 T cells:GM-CSF^{+}; (2) IL-17^{+}GM-CSF^{+} Th cells:GM-CSF^{+}IL17A^{+}GPR65^{high} ; (3)IFN-γ^{+}GM-CSF^{+}Th cells:GM-CSF^{+}IFN-γ^{+}	IL-7,GPR65				(1)IL-7/naïve CD4+T cells/GM-CSF(promote);(2)GPR65/ IL-17^{+}GM-CSF^{+} Th cells/GM-CSF(Promote)	PMID: 29142230	2017	Unique transcriptome signatures and GM-CSF expression in lymphocytes from patients with spondyloarthritis
Psoriasis(mouse)	GM-CSF $^{+}$ CD4^{+} T cells	CD45^{+}GM-CSF^{+}IL-23R^{+}IL-22^{-}IL-17A^{-} IL-17F^{-}			IL-1β; IL-2	Treg	(1)IL-2/CD4+T cells/GM-CSF(Pro); (2) IL-23/IL-23R/CD4+T cells /GM-CSF(Pro); (3)IL-1β/CD4+T cells /GM-CSF(Pro)(4)Treg- CD4+T Ccells/GM-CSF(Inh)	PMID:30590032	2018	Identification of functional enhancer variants associated with type I diabetes in CD4+ T cells

Cells in vitro	GM-CSF-producing T cells	GM-CSF^{+}IL-3^{+}			Rel		Rel/NF-κB/GM-CSF+Th cells/GM-CSF(Pro)	PMID: 8622948	1996	Rel-deficient T cells exhibit defects in production of interleukin 3 and granulocyte-macrophage colony-stimulating factor
Cells in vitro	ThGM cells	GM-CSF^{+} IFN-γ^{-}IL-4^{-} IL-17^{-} CD45^{low}CD95^{high}CD69^{high}T-bet^{-} GATA-3^{-}RORγt^{-} FoxP3^{-}NFATc1+AP-1+ RUNX-1+			GM-CSF, IL-2	IFN-γ; IL-4; IL-12	IL-2 or GM-CSF/naïve CD4+ T cells/GM-CSF（Pro）	PMID:24076588	2013	A novel subset of helper T cells Pros immune responses by secreting GM-CSF
DED(dry eye disease)	GM-CSF–producing Th17 cells	GM-CSF^{+}TNF-α^{+}IL-17A^{+}IL-17F^{+}IL-22^{+}CCL20^{+}						PMID:28241321	2017	T Cell–Derived Granulocyte-Macrophage Colony-Stimulating Factor Contributes to Dry Eye Disease Pathogenesis by Promoting CD11b^{+} Myeloid Cell Maturation and Migration
EAU(Experimental autoimmune uveitis)(mouse)	GM-CSF-producing CD4^{+} T cells	GM-CSF^{+}						PMID:32593472	2020	Autoimmunity to neuroretina in the concurrent absence of IFN-γ and IL-17A is mediated by a GM-CSF-driven eosinophilic inflammation

GN(Glomerulonephritis)	GM-CSF-producing Th1 cells（GM-CSF^{+} T cells）	GM-CSF^{+}IFNγ^{+}IL-17A$^{+(few)/-}$IL-22$^{+(few)/-}$IL-10$^{+(few)/-}$						PMID: 36921033	2023	CD4+ T cells produce GM-CSF and drive immune-mediated glomerular disease by licensing monocyte-derived cells to produce MMP12
endometriosis(mouse)	ThGM cells	IL-35R-GM-CSF^{+} IL-2^{+}TNF-α^{+}IL-3^{+} IL-17$^{+/-}$IFN-γ^{-}IL-4^{-}T-bet^{-}GATA3^{-}RORγt^{-}IFN-γ^{-}IL4^{-} CCL20+CXCR3^{-}CCR8^{-}CCR4^{+}CCR10^{+}			IL-1β； IL-7		IL-1β or IL-7/naiveCD4+ T cell/GM-CSF(Pro)	PMID:37944204	2023	Differential expression of interleukin-35 receptor distinguishes different subsets of granulocyte-macrophage-colony-stimulating factor-producing T helper cells in a mouse endometriosis model
GVHD（mousse）	IL-7RhiGM-CSF+ T cells	GM-CSF^{+}IL-7R^{+} IFN-γ$^{+/-}$TNF-α$^{+/-}$			BATF; IL-7; STAT5; GM-CSF		（1）IL7/IL-7R/BATF/IL-7RhiGM-CSF+ T cells/GM-CSF（Pro）；（2）STAT5/IL-7RhiGM-CSF+ T cells or naïve CD4+ T cells/GM-CSF（Pro）；（3）GM-CSF/ naïve CD4+ T cells/GM-CSF（Pro）	PMID:29376889	2018	BATF-dependent IL-7RhiGM-CSF+ T cells control intestinal graft-versus-host disease
GVHD(human andmouse)	GM-CSF + T cells	GM-CSF+IFN-γ+（50%）/-IL-17-						PMID:30487251	2018	Graft-versus-host disease, but not graft-versus-leukemia immunity, is mediated by GM-CSF-licensed myeloid cells

GVHD	GM-CSF-secretingcells	GM-CSF^{+}IL-17$^{+/-}$BATF^{+}			BATF		BATF/GM-CSF+ Th cells or naïve CD4+ T cells/GM-CSF（Pro）	PMID: 31585949	2019	Donor T-cell–derived GM-CSF drives alloantigen presentation by dendritic cells in the gastrointestinal tract
GVHD(mouse)	Bhlhe40+GM-CSF+CD4+ T cells	GM-CSF^{+}Bhlhe40^{high} α4β7^{high}CCR9^{high}CD11C^{+}			Bhlhe40		Bhlhe40/CD4+T cells/GM-CSF	PMID:27619472	2020	Pathogenic Bhlhe40+ GM-CSF+ CD4+ T cells Pro indirect alloantigen presentation in the GI tract during GVHD
GVHD(mouse)	CD4^{+}GM-CSF^{+}T cells:(1)CD4^{+}GM-CSF^{+}IFNγ^{+}T cells; (2) CD4^{+}GM-CSF^{+}IFNγ^{-}T cells	(1)CD4^{+}GM-CSF^{+}IFNγ-T cells (GM-CSF^{+}IFNγ-TNF-α+GATA^{high}T-bet^{low}ST2^{high}CD69^{high}PD-1^{low}LAG3^{low}); (2)CD4^{+}GM-CSF^{+}IFNγ+T cells(GM-CSF^{+}IFNγ-TNF-α^{+}T-bet^{high})			IL-2; STAT5; IL-7/IL-7R		(1)IL-2/STAT5/naïve CD4+ T cells/GM-CSF(Pro); (2)IL-7/ IL-7R/STAT5/naïve CD4+ T cells/GM-CSF（Pro）	PMID: 35015822	2022	Single-cell immune profiling reveals a developmentally distinct CD4^{+} GM-CSF^{+}T-cell lineage that induces GI tract GVHD
